# Improving access to endogenous DNA in ancient bones and teeth

**DOI:** 10.1038/srep11184

**Published:** 2015-06-17

**Authors:** Peter B. Damgaard, Ashot Margaryan, Hannes Schroeder, Ludovic Orlando, Eske Willerslev, Morten E. Allentoft

**Affiliations:** 1 Centre for GeoGenetics, Natural History Museum, University of Copenhagen; 2 Faculty of Archaeology, Leiden University, PO Box 9515, 2300 Leiden, The Netherlands

## Abstract

Poor DNA preservation is the most limiting factor in ancient genomic research. In the majority of ancient bones and teeth, endogenous DNA molecules represent a minor fraction of the whole DNA extract, rendering shot-gun sequencing inefficient for obtaining genomic data. Based on ancient human bone samples from temperate and tropical environments, we show that an EDTA-based enzymatic ‘pre-digestion’ of powdered bone increases the proportion of endogenous DNA several fold. By performing the pre-digestion step between 30 min and 6 hours on five bones, we observe an asymptotic increase in endogenous DNA content, with a 2.7-fold average increase reached at 1 hour. We repeat the experiment using a brief pre-digestion (15 or 30 mins) on 21 ancient bones and teeth from a variety of archaeological contexts and observe an improvement in 16 of these. We here advocate the implementation of a brief pre-digestion step as a standard procedure in ancient DNA extractions. Finally, we demonstrate on 14 ancient teeth that by targeting the outer layer of the roots we obtain up to 14 times more endogenous DNA than when using the inner dentine. Our presented methods are likely to increase the proportion of ancient samples that are suitable for genome-scale characterization.

With the introduction of next-generation sequencing (NGS) technology, ancient DNA (aDNA) research has advanced over the last decade from retrieving short segments of mitochondrial DNA (mtDNA) to the characterization of complete genomes (reviewed in^1^). Among many highlights in such paleogenomic research is the documentation of a Late Pleistocene admixture between Neanderthals and anatomically modern humans^2^,^3^, the discovery of a previously unknown hominin, the Denisovan^4^,^5^,^6^, the description of the early human colonisation of the Americas and the Arctic^7^,^8^,^9^,^10^ and the clarification of equine evolution by sequencing the complete genome of a 700,000 years old horse^11^.

With some exceptions^12^,^13^, the ancient hominin samples subjected to genomic sequencing have displayed an exceptional biomolecule preservation with 28–70% of the DNA in the extracts identified as authentic^3^,^5^,^7^,^9^,^14^,^15^,^16^,^17^,^18^. However, this proportion of authentic DNA molecules, referred to as the ‘endogenous’ DNA content, represents less than 1% in the vast majority of DNA extracts from ancient remains (e.g.,^19^,^20^). Instead, the bulk of the DNA content in aDNA extracts is normally microbial^21^,^22^. Obtaining whole genomes, or genome-wide information is preferentially achieved using Next Generation ‘shotgun’ Sequencing, but when the target molecules represent such a minute fraction this is either not feasible or, at best, extremely expensive. Although genomic capture methods can be used to enrich for the target DNA^19^, most ancient samples will remain unsuitable for genome-scale characterization. It is therefore common to extract a large number of samples in an initial screening phase and then shotgun sequence them at low depth, in order to identify a few good candidate samples that have high endogenous contents. This approach is time-consuming and expensive, and requires the destruction of many samples that will not be amenable to genome analyses. Thus, the field of paleogenomics will benefit greatly from methodological advances that result in an increase of the endogenous DNA fraction during the DNA extraction. In this study we present two such advances.

Although the biochemical processes driving DNA preservation in bone are not fully understood, it has been shown that aDNA is preserved both in association with the bone minerals, hydroxy-apatite aggregates, and within the organic collagen fibrils^23^,^24^. During decomposition the bone structure degrades, which increases the porosity and total surface area of the bone^23^,^25^. Although the colonization of microorganisms is likely heterogenous^26^, we expect that during the digestion of bone material in the first step of an aDNA extraction, surface contaminants will be released into solution first, regardless of their exact location of deposition. This is in contrast to the endogenous DNA which is likely located more protected within the bone’s microniches^27^. We therefore hypothesized that treating the grinded bone material with a digestion buffer for a short period of time (a “pre-digestion”) would remove a fraction of the exogenous non-target DNA, and thereby enrich the DNA extract for endogenous DNA. Similarly, we hypothesized that modern human DNA contamination, deposited on the bone surface during recent handling, would also be preferentially removed with such pre-digestions. Higher endogenous DNA fractions were recently observed on second extractions undertaken on remaining bone pellets that had not been fully dissolved after 24 hours of incubation in a digestion buffer^22^,^27^,^28^. These promising observations provided an impetus for a more systematic assessment of the phenomenon in order to validate the potential for implementing a pre-digestion step into standardized aDNA extraction protocols, and identify the optimal duration of a pre-digestion.

We therfore used Next Generation shotgun sequencing to monitor the changes in endogenous DNA content and sequence complexity in DNA extracts from bone material treated with varying pre-digestion times (30 mins to 6 hours). Four bones from Easter Island (post-AD 1200), and one bone from Copenhagen, Denmark (18^th^ century) were included in this initial experiment. Additionally, 21 ancient bones and teeth from Easter Island (post-AD 1200), Hungary (Bronze-Age 2000-1500 BC), and Guadeloupe (400-1400 AD) were used in a follow-up experiment to confirm the efficiency of the method and test the significance of the improvement when applying brief pre-digestions (i.e. 15–30 minutes).

Teeth roots have been demonstrated as an excellent resource for aDNA^29^,^30^,^31^ but a systematic comparison of the endogenous DNA proportions in various parts of a root is lacking. It has been shown that the nuclear DNA concentrations decline drastically in the inner dentine layer throughout the life of an individual^32^, whereas levels of nucleated cells in the apical cementum layer are unaffected by age^31^ . Moreover, a quantitative PCR approach determined that the concentration of human mtDNA in ancient teeth is generally elevated in the cementum as compared to the dentine^29^. However, because the cementum layer is exposed at the root surface, it could potentially be more affected by microbial colonization than dentine, and would thus still display a lower endogenous DNA proportion. To investigate this we extracted DNA and used shotgun sequencing to estimate the endogenous DNA proportions in crushed root surface (likely to be enriched for the outermost cementum layer), and the deeper parts of the root (containing mainly dentine) from 14 ancient teeth (Table 1), from Denmark (18^th^ century and Iron Age c. 100 AD), Easter Island (post-AD 1200), and Greenland (c. 1100 AD).

## Materials and methods

All the laboratory work was performed in the dedicated clean laboratory facilities at the Centre for GeoGenetics, Natural History Museum, University of Copenhagen, according to strict aDNA standards^33^,^34^.

### Sample information

A total of 26 ancient human bones and teeth from various archaeological contexts spanning tropical and temperate environments were included in the pre-digestion experiments. Fourteen ancient teeth were used in the comparison between DNA extracted from the root core (dentine) and root surface (cementum-enriched). All relevant information regarding the samples are provided in Table 1.

### DNA extractions with varying pre-digestion time

**Figure 1:**
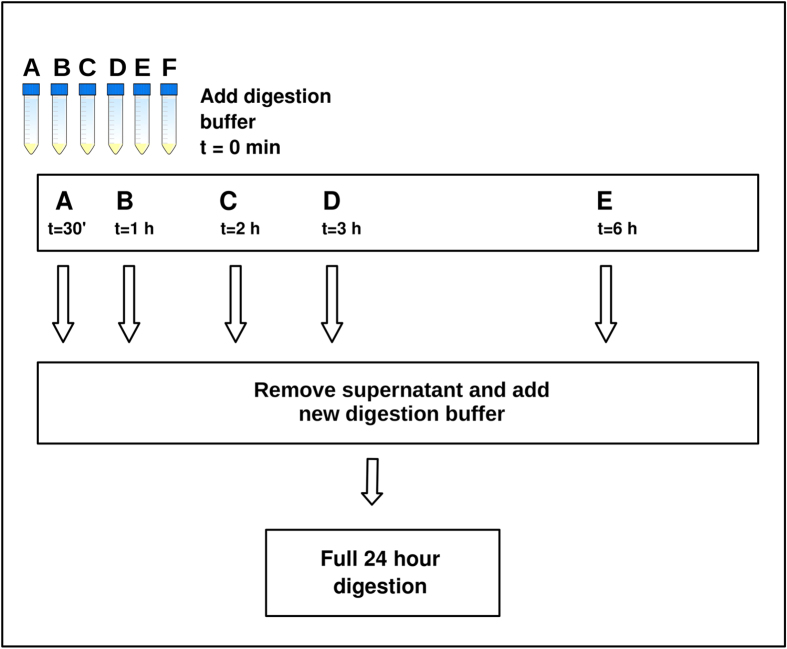
Pre-digestion experiment. For each of five bones, c. 2.5 g of bone powder was homogenized and 400 mg distributed into 6 tubes labelled A–F. A digestion buffer was added to all samples at time (t) = 0 and samples were vortexed and left on rotator at 50 °C. At t = 30 minutes, the pre-digestion was removed from sample A and a new digestion buffer was added, followed by a full 24-hour incubation. Similarly, pre-digestions were removed from samples B–E at respectively 1 hour, 2 hours, 3 hours, 6 hours. No pre-digestion was removed from sample F.

The bone surface at the sampling area was removed using a scalpel or a sterile drill bit. Cortical bone mass was drilled and homogenized, and 400 mg of bone powder was transferred to each of six 15 mL Falcon tubes (labelled A–F) (Fig. 1). To counteract the effect of granular convection by which the smallest bone particles end up in the first tubes, we homogenized the powder between each transfer.

The six sub-samples were subjected to a digestion buffer containing 4.7 mL 0.5 M EDTA, 50 μL recombinant Proteinase K, and 250 μL 10% N-Laurylsarcosyl and incubated simultaneosuly at 50 °C. After 30 minutes, extraction A was centrifuged and the supernatant (the pre-digest) was removed. An identical digestion buffer was then transferred to the undigested and sedimented bone powder, and the sample was vortexed and returned to incubation for a full 24 hour digestion (Fig. 1). Similarly, pre-digest supernatants were removed for extractions B-E at time points: 1 hour, 2 hours, 3 hours, 6 hours respectively and new digestion buffer added. No pre-digest supernatant was removed for extraction F. At 24 hours all digestions were stopped (Fig. 1). The samples were then centrifuged and the supernatants transferred to new tubes for DNA extraction. Five bones (Table 1) were selected for this initial experiment aiming to optimize pre-digestion time, yielding a total of 30 DNA extracts.

A guanidinum thiocyanate-based binding buffer^11^ was used when extracting the DNA from the supernatant. The buffer was prepared by mixing 118.2 g Guanidinium Thiocyanate with 10 mL Tris 1 M, 1 mL NaCl 5 M, 8 mL EDTA 0.5 M, 1 g N-Lauryl-Sarcosyl and water to a total volume of 200 mL. 20 mL of the binding buffer was transferred to each sample and left rotating for 3 hours with 100 ul silica powder in solution to bind the DNA. After DNA-binding, the silica was centrifuged and washed twice with 1 ml 80% cold ethanol, and the DNA eluted in 80 μl EB Buffer (Qiagen). The DNA concentration in all final extracts was measured using Qubit® Fluorometric Quantitation (Life Technologies, Grand Island, NY).

Following this initial experiment, we tested the consistency of the improvement with short pre-digestion times on 17 ancient and historical bone samples from Easter Island, Guadeloupe and Hungary (Table 1) and 4 ancient teeth from Guadeloupe. Each drilled bone sample was homogenized and split into two equal amounts, one that was extracted with 15 or 30 minutes of pre-digestion treatment, and one that was not pre-digested. The roots of the four teeth were drilled into powder post-removal of the inner dentine and likewise split into two fractions. The extraction procedure was identical to the one described above.

**Figure 2:**
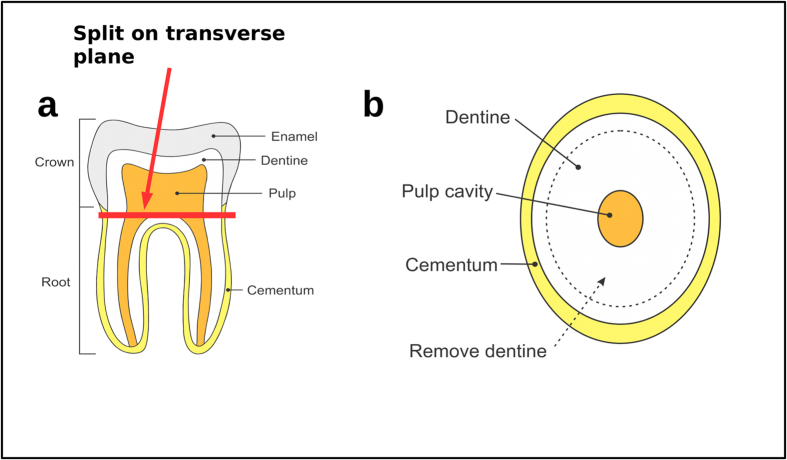
Sampling a tooth. (**a**) The tooth is split on the transverse plane using a cutting disk, (**b**) the dentine inside the root is removed creating a hollow root cap that is likely to be enriched for cementum. The root cap is crushed and used for DNA extraction, while the dentine can serve as adequate substrate for stable isotope analysis or radiocarbon dating.

In an additional experiment we used 14 ancient teeth (Table 1) to compare the endogenous DNA content of root surface against the inner dentine core. First, the outermost surface of the teeth was removed with a drill-bit or a scalpel, as is standard procedure to exclude the most obvious surface DNA contamination. The effect of the cleaning was observed as a change in surface color, but it was done gently and did not result in a visible effect on the thickness of the root. Next, each tooth was split with a cutting disk into two pieces (the crown and the root) on the transverse plane (Fig. 2). The dentine was then drilled out of the root from the pulp cavity and transferred to a sterile tube, leaving a hollow ‘root cap’. This root cap, likely to be enriched for cementum, was then crushed with a mortar or cut into smaller pieces before being transferred to a sterile tube. The two fractions from each tooth root (crushed root surface and drilled dentine core) were then extracted separately as above, but without pre-digestion.

### Library preparation and sequencing

Blunt-end Illumina sequencing libraries were built following guidelines previously outlined^11^, using the NEBNext® DNA Library Prep Master Mix Set E6070 (New England Biolabs Inc., Manual Version 2.1^35^. The libraries were amplified using a two-round PCR setup^9^,^36^ with primers containing a 6 bp known index sequence. Details on the library preparation and PCR amplification conditions can be found in Supplementary Information Methods S1.

The amplified libraries were quantified on an Agilent 2200 TapeStation (Agilent Technologies, Palo Alto, CA, USA) or an Agilent Bioanalyzer 2100. The library pools were sequenced (100 bp, single read) at the Danish National High-throughput DNA Sequencing Centre. Basecalling and sequence sorting by sample-specific indexes was performed by the Sequencing Centre using CASAVA v.1.8.2.

### Data analyses

All reads were trimmed for adapter sequences using AdapterRemoval 1.5.2^37^, and only reads with a minimal length of 30 bp were retained. The trimmed sequences were mapped against the human reference genome Hg19, HS Build37.1, using bwa^38^ with the *samse* function using standard parameters except that seeding was disabled, following published recommendations^39^. We used all the sequences that mapped uniquely to one position in the human reference genome and then removed duplicate sequences from the output bam file using the *rmdup* function in samtools^40^. The relevant summary statistics (Supplementary Tables S1-S3) used to estimate the endogenous DNA fraction (fraction of uniquely mapped human sequences divided by the total number of sequences passing trimming) and sequence clonality (proportion of duplicate human sequences), were extracted with a custom Perl script. The clonality of each library will increase with increased sequencing depth, implying that the overall sequencing efficiency (fraction of non-duplicated endogenous DNA sequences divided by total sequences) decreases. Hence we down-sampled the raw sequencing files (fastq) to match the smallest number of sequences per bone to allow for a direct comparison of sequencing efficiency with or without the new extraction methods.

We also investigated the data for signatures of DNA damage. This was done in part to confirm that the profiled human DNA was not modern contamination, and in part to measure if the pre-digestion treatment would result in any obvious compositional biases in the DNA, or damage it further.

Based on the sequence length distributions of the sequences identified as human, we estimated the decay constant *k* (representing the fraction of broken bonds in the DNA backbones) and average DNA fragment length in the extract (1/*k*), as previously described^41^,^42^. A large *k* value reflects a pronounced exponential accumulation of small DNA fragments as a consequence of *post mortem* DNA breakage which is a signature of highly degraded DNA. Following the approach described in^42^, we investigated only the declining part of the distribution for each sample (40–90 bp) since the ends of the distribution are biased respectively by poor recovery of <40 bp fragments during the DNA extractions (and the library building process), and the accumulation of >94 bp reads sequenced to the maximum length on the Illumina platform with the here applied chemistry.

Using standard parameters in the Bayesian approach implemented in mapDamage 2.0^43^ we estimated the position-specific cytosine deamination probability (δs) as well as the probability of a base being positioned within a single-stranded overhang (λ), which thus relates directly to the average length of the overhangs, reflecting *post mortem* damage of aDNA^35^. In order to increase the accuracy of the damage estimates performed on the ancient human DNA fractions, these were based on the total mapped datasets and not the downsampled files. Outputs from mapDamage 2.0 were analysed and plotted with R.

Sequence quality control statistics were generated using fastqc on the retrieved human sequences from each (not downsampled) file, as well as the total sequences of the library (downsampled human + non-human sequences), and were used to check for abnormalities, and particularly to investigate for potential changes in GC-content following pre-digestion. The results were plotted with R.

Despite the implementation of strict aDNA protocols, it is difficult to completely avoid contamination from modern DNA when working with ancient human material - in particularly when dealing with samples that have been handled previously during excavation and while stored at museum collections^44^. It was therefore important to establish that any potential increase in endogenous DNA content following our treatments was not an effect of DNA contamination. Accurate estimates of contamination levels require large amounts of genomic data, so we here restricted this analysis to samples with a mitochondrial genome coverage above five times (5X). We used contamMix^45^ (Fu *et al.*, 2013) to estimate the level of human DNA contamination in the mtDNA sequences. This method compares for each individual the mapping affinities of its mtDNA sequences to its own consensus mitogenome sequence, relative to the mapping affinity of its mtDNA sequences to a dataset of potential contaminants represented by 311 mitogenomes from worldwide populations. The mitogenome consensus sequences were made using the samtools *mpileup* function (Li *et al.*, 2009) and filtering the variant list outputted with bcftools with a script previously used (Jacobsen *et al.*, 2014), selecting only bases with a coverage >5X and >50% concordance between bases, in order not to incorporate bases with only limited depth-of-coverage and minimize biases from sequencing errors and DNA damage misincorporations. One sample, Rise479 A, displayed heavy contamination at informative sites (ie. ~50%) which prevented the determination of a reliable consensus sequence, thus challenging the contamination assessment. However, a sample from the same individual Rise479 B (the pre-digested extract from the same bone) revealed only minimal contamination (ie. 1.2%). We could therefore use the consensus sequence obtained from this extract as a representation of the true mitochondrial sequence of Rise479 A, and thereby produce a reliable contamination estimate in the latter (see Table 2 for results).

## Results

### Effects of pre-digestion time

Following 24 hours of digestion, the samples displayed a spectrum of colors, in which long pre-digestion times resulted in lighter colors of the final digest, likely attributable to the removal of dirt and contaminant particles with long pre-digestion treatments. This observation is accompanied by a decline in total DNA concentrations in the extracts as a function of pre-digestion time (Supplementary Fig. S1). In three cases, the DNA concentration as a function of pre-digestion time is described well by an exponential decline (R^2^ values 0.72–0.99), while the fit is more modest in two cases (R^2^ = 0.40 and 0.63) (Supplementary Fig. S1).

**Figure 3:**
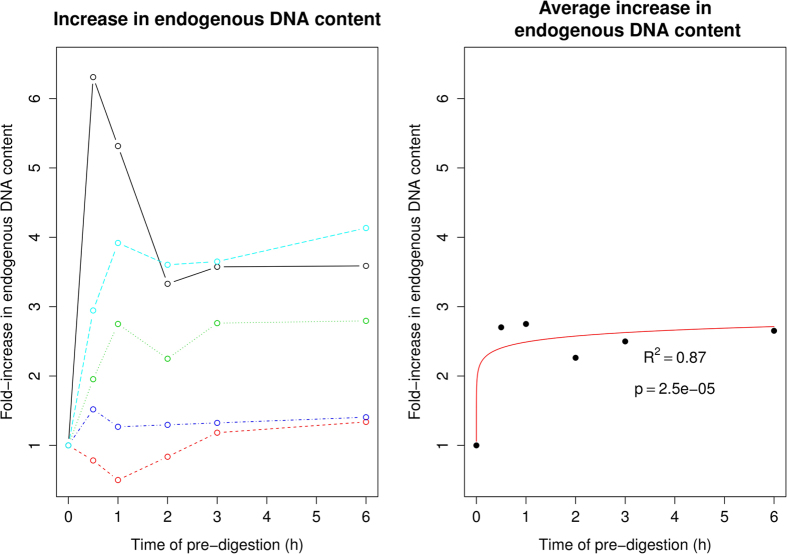
Effect of length of pre-digestion time. The graph represents fold-increase in endogenous DNA content according to pre-digestion lengths. (**A**) Fold-increase in endogenous DNA content in sample EI8 (red), EI9 (green), EI19 (blue), EI22 (cyan), Trinitatis (black). (**B**) A logarithmic model fitted to the mean increase suggests an asymptotic growth (p = 2.5 e-5).

By aligning an average of ~17 million reads per library against the human reference genome Hg19, we tracked the endogenous DNA content as a function of pre-digestion time. Despite very low endogenous DNA contents (0.001% to 1.6%, see Table 1) the pre-digested extracts showed an increase in human DNA (Fig. 3), with an average fold-increase of c. 2 after 30 minutes of pre-digestion and c. 3 after 1 to 6 hours of pre-digestion. Despite sample to sample variation, and some extreme outlier values, the average increase in endogenous DNA content following pre-digestion appeared to be logarithmic (R^2^ = 0.87, p = 2.5 e-5) reaching the asymptotic maximum after c.1 hour of pre-digestion (Fig. 3).

Because DNA concentrations decrease with longer pre-digestion treatments, we tested if this could be tracked as an increase in sequence clonality among the human DNA sequences due to endogenous DNA loss. An increase in clonality would result in poor library sequencing efficiency despite a higher endogenous DNA content. Overall, clonality levels were low with average values ranging from 1.5% (no pre-digestion) to 4.8% (6 hours of pre-digestion) (see Supplementary Table S1), although we note that library complexity predictions from small datasets of shallow sequencing can give false estimates of library complexity (Daley and Smith, 2013). For bone EI8 the clonality increased from 1.3% (no pre-digestion) to 18% (6 hours of pre-digestion) causing a complete stagnation in the increase in library efficiency (see Supplementary Table S1).

### Brief pre-digestions on 21 samples

**Figure 4:**
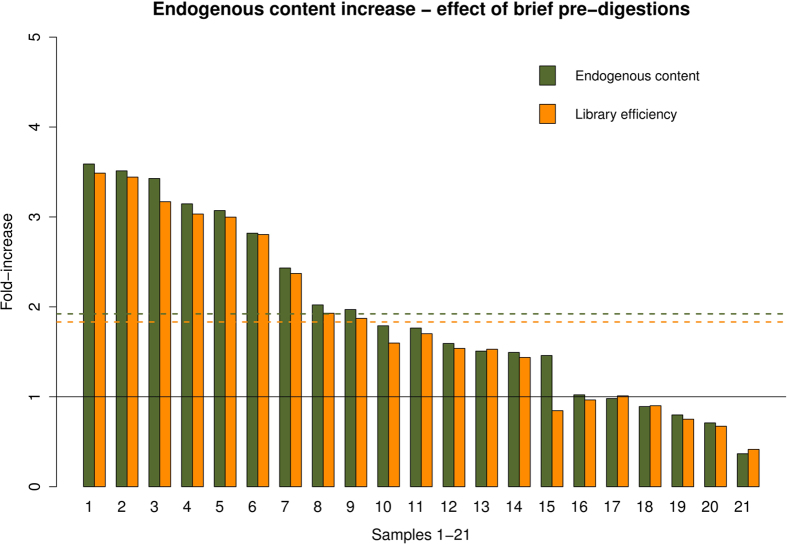
Brief pre-digestions. The graph represents the endogenous content increase following a brief pre-digestion (15 or 30 minutes). Green bars represent the increase in endogenous content, and yellow bars represents the increase in library efficiency. Dashed lines represent mean values. 16 samples show higher endogenous content (fold-increase higher than 1, black line) when pre-digested an the overall increase is significant as revealed by the one-side t-test: (t = 2.56, df = 20, p-value = 0.009). Samples 1–21 are 1) AAG6, 2) AAG8, 3) ANR 4709, 4) AAG9, 5) AAG7, 6) ANR 4714, 7) AAG2, 8) AAG5, 9) AAG4, 10) Rise 479, 11) ANR 4704, 12) AAG1, 13) AAG3, 14) ANR 4705, 15) Rise 483, 16) ANR 4707, 17) ANR 4708, 18) ANR 4715, 19) ANR 4706, 20) ANR 4710, 21) ANR 4712.

Given that short pre-digestion times appeared to improve access to endogenous DNA content, we next compared the endogenous human DNA content of 17 bones and 4 teeth extracted with and without a brief pre-digestion of 15 or 30 minutes (Table 1), in order to confirm the efficiency of the method. The mean enrichment in endogenous DNA content was c. 2-fold (Fig. 4), and highly significant as revealed by a one-sided paired t-test (t = 2.56, df = 20, p-value = 0.009).

The overall sequencing efficiency increase was similar to the increase in endogenous DNA content, reflecting that clonality levels were nearly equivalent in the pre-digested and the non-pre-digested samples. Only one sample (Rise483) displayed a clear loss of sequence complexity when pre-digested (Fig. 4, Sample 15 and Supplementary Table S2) resulting in lower library efficiency (140353 normalized non-clonal human reads) compared to the non-pre-digested sample (165954 normalized non-clonal human reads).

### Effects of pre-digestion on DNA composition

In general we observed a negligible change in the GC-content in the total datasets (human + non-human) following pre-digestion (Supplementary Fig. S3). The genomic GC-content in the identified human reads was ~50% for AmpliTaq Gold amplified libraries and ~40% GC for Kapa U + amplified libraries, but there was no correlation between GC-content in the human fraction and pre-digestion times (Supplementary Fig. S2).

Similarly, the DNA damage parameters δs and λ for the human reads displayed no general trend as a function of pre-digestion time and likewise no general pattern was discernible for the decay constants (*k*) and the estimated average fragment length (1/*k*) of the human DNA in the extracts (Supplementary Figure S4-S6, Supplementary Table S4).

### Human DNA from the dentine core and cementum-rich surface of teeth roots

**Figure 5:**
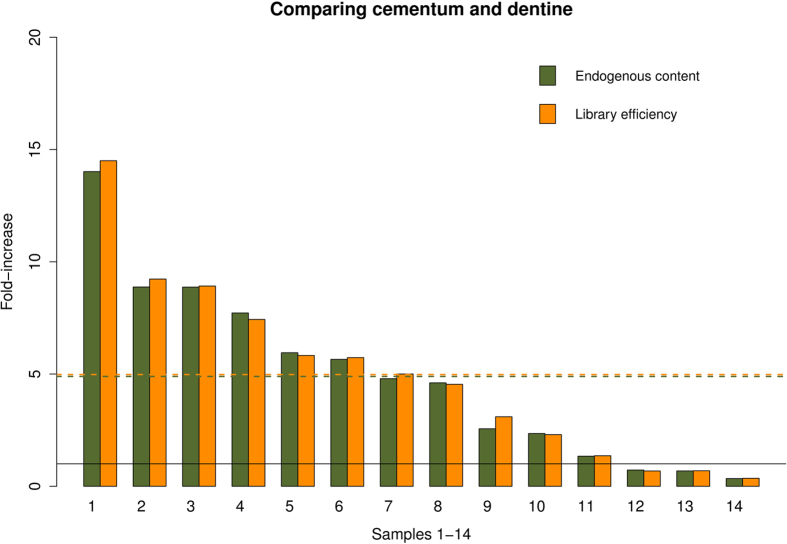
Endogenous content in root surface and dentine. Green bars represent fold-increase in endogenous content when sampling from the root surface instead of the dentine from the same tooth. Yellow bars represent fold-increase in library efficiency. Dashed lines is the average fold-increase. Eleven samples show higher endogenous content (fold-increase higher than 1, black line) in the tooth surface compared to the dentine, and the average increase is significant as revealed by the one-side t-test: (t = 2.10, df = 13, p-value = 0.05). Sample 1–14 are 1) VHM00500 × 73, 2) VHM00500 × 81, 3) ID-530, 4) VHM00500 × 7, 5) VHM00500 × 77, 6) ANR 4709, 7) VHM00500 × 22, 8) ANR 4711, 9) ID-532, 10) Trinitatis 2, 11) ID-677, 12) ID-678, 13) ANR 4713, 14) Trinitatis 1.

Finally, we investigated whether the outer layer of teeth roots contained higher endogenous DNA proportions than the dentine, which represents the inner part of the tooth. For 11 of 14 teeth, we observed a higher fraction of human DNA in the root surface when compared to the dentine (Fig. 5). The mean fold-increase in endogenous DNA proportion was c. 5-fold with values ranging from 0.3-fold to 14-fold. The one-sided paired t-test reveals a significant increase in endogenous DNA at the surface as compared to the dentine core (t = 2.10, df = 13, p-value = 0.05).

The library efficiency increase was almost identical to the endogenous DNA proportion increase (Fig. 5), signifying that extracting from the root surface will generally yield a higher proportion of human DNA without compromising complexity among the template molecules. Finally we note that in 11 out of 14 teeth the cytosine deamination ratio (δs) was significantly elevated in the dentine as compared to the cementum-enriched surface (Table 3).

### Ancient DNA authentication

For all DNA extracts we observed elevated δs and λ values (see Supplementary Fig. S6) suggesting that the bulk of the DNA templates are of ancient origin. The level of DNA contamination was investigated for 12 extracts where we had sufficient data to meaningfully conduct this analysis (Table 2). Contamination levels proved negligible in 10 extracts, confirming that our observed enrichment in endogenous DNA was not driven by modern human contaminant DNA. Encouragingly, in two bone samples the pre-digestion resulted in reduced contamination levels from 45% to 1.2% in the Rise479 sample, and from 2.5% to 0.3% in Rise483. Likewise, while tooth VHM00500 × 81 appeared contaminated in the dentine fraction, contamination levels in the cementum fraction was estimated to be only 0.1% (Table 2).

## Discussion

### Pre-digestion time

This study has documented several improvements of immediate value to aDNA research. We show that pre-digestion is a simple and effective means to remove a proportion of non-target DNA from ancient samples. With an average value of 2.7-fold enrichment of the endogenous DNA content following 1 hour of pre-digestion, one would ultimately generate 2.7 times more usable data for the same price.

We interpret the asymptotic increase with longer pre-digestion treatments as a gradual change in the ratio of dissolved exogenous DNA over endogenous DNA. Although the endogenous DNA fraction increases with longer pre-digestions, the benefits of waiting this long are likely to be marginal.

We expected to observe an increase in DNA sequence clonality following longer pre-digestion times because of two complementary phenomena, namely that i) we have sequenced the human DNA fraction deeper with the same sequencing effort because exogenous DNA is now reduced, and/or ii) we have sequenced the human fraction deeper because there has been a significant reduction in the DNA library complexity - the human fraction included. The first phenomenon would be a positive outcome of pre-digestion, as it simply requires less sequencing effort before saturation is reached, similar to genomic capture methods^19^. The second phenomenon is problematic as it would reflect a loss of sequenceable genomic material. With long pre-digestion times we find saturation in the increase of endogenous DNA content, accompanied with a loss in DNA concentration (see Supplementary Fig. S1). In one case, we observe a considerable increase in sequence clonality. We deduce from these observations that it is advisable to apply this method conservatively using short pre-digestion times (15–30 mins).

We also stress that the optimal time of pre-digestion will depend on the temperature used during incubation. Here we have incubated at 50 °C, but if incubating at lower temperatures, it may be advantageous to apply a longer pre-digestion step.

### Changes in genomic composition and DNA damage

It is conceivable that a pre-digestion treatment could result in DNA damage patterns different from those in non-pre-digested samples, either because the pre-digestion itself would induce more DNA damage or because it would increase accessibility to human DNA molecules in subniches with different preservation conditions. However, for the pre-digestion lengths tested in this study, we find no trends on the estimated DNA damage parameters or GC-contents (Supplementary Fig. S2, S4–S6, Supplementary Table S4). These observations provide evidence that the pre-digestion procedure will not damage the DNA or otherwise alter the genomic composition. This could be because the pre-digestions used in these experiments are relatively brief (<6 hours) and unspecific in dissolving both the organic and mineral phases of the samples. Hence they may not provide a basis for comparing endogenous DNA preserved within different subniches, such as that analyzed in Schwarz *et al.*, (2009).

### Comparing DNA in the surface and dentine core of teeth roots

In 11 of 14 samples we observe an increase in endogenous DNA content when extracting DNA from the hollow root cap compared to the drilled-out dentine. This demonstrates that the root surface is a highly advantageous substrate for aDNA extractions and we propose two possible explanations for this observation.

1. The thin cementum layer is located near the surface of the roots as shown on Fig. 2. Although we remove the external outermost surface in the initial sample preparation and cannot remove all of the dentine from the root cap, it seems highly likely that we enrich the sample for cementum with this method. Cementum has previously been shown to contain a higher concentration of human DNA compared to dentine^29^ and our results suggest that this is manifested in a higher proportion of human DNA reads in the total extract. Moreover, our DNA damage assessments determined that the human DNA preserved in the inner dentine displayed higher cytosine deamination ratios as compared to the DNA preserved in the cementum-enriched fraction, indicative of differential preservation conditions. While these observation are not necessarily related to altered access to bacterial invasion and hence endogenous levels, it underlines the impact of histological differences in DNA preservation.
2. The dentine is in direct contact with the pulp cavity (Fig. 2). With the traditional dentine drilling method it is therefore not unlikely that exogenous microbial DNA from the pulp cavity is co-extracted with the dentine, which then translates into a lower human DNA content in the sequencing.

The results of the contamination analyses (Table 2) showed that the elevated fraction of human DNA in the outer root layers is not simply due to DNA contamination. When teeth are available for aDNA studies, the results presented herein strongly support targeting the cementum-rich root surface. As a side note, using only the root of a tooth for the aDNA extraction facilitates leaving the crown intact, which can then be used for morphological analyses. The removed dentine remains a suitable material for stable isotope analyses or radiocarbon dating.

## Recommendations and conclusion

We recommend following these five points when extracting aDNA from ancient bone or teeth:

1. Apply a brief pre-digestion step (15–30 mins).
2. If sufficient material is available, then run several extractions in parallel with differing pre-digestion lengths.
3. Do not discard the supernatant (the pre-digest) at first, as it will contain a fraction of endogenous DNA molecules.
4. Do not discard undigested bone pellets post-24-hour digestion, as they are likely to contain a higher endogenous DNA fraction than the first extraction (whether this was pre-digested or not).
5. Sample the surface of teeth roots in favor of the inner dentine but remove the outermost surface layer to minimize the risk of including DNA contamination.

We advocate caution when implementing a pre-digestion step if only a small amount of sample material is available (i.e. <50 mg). In such cases it is not recommendable to pre-digest the sample because the DNA concentration in the final extract may become critically low. Finally, we note that if pre-digestion is combined with other methods that have demonstrated an enrichment in the endogenous DNA fraction, such as the capture of mitogenomes^46^ or whole genomes^19^, single-stranded sequencing libraries^47^, or damage-enriched single-stranded sequencing libraries^48^, it is likely to result in a many-fold increase in the endogenous DNA proportion. We believe that an implementation of the simple and inexpensive procedures we have here described will increase the discovery rate of ancient samples that are suitable for genomic research, but the methods should prove equally useful for other disciplines working with degraded DNA such as forensic sciences.

## Additional Information

**How to cite this article**: Damgaard, P. B. *et al.* Improving access to endogenous DNA in ancient bones and teeth. *Sci. Rep.* **5**, 11184; doi: 10.1038/srep11184 (2015).

## Supplementary Material

Supplementary Information

## Figures and Tables

**Table 1:** Sample information

Experiment type	Sample name	Origin	App. age	Sample type
Pre-digestion time	EI9	Easter Island	Post-1200 AD	Long bone
	EI19	Easter Island	Post-1200 AD	Long bone
	EI22	Easter Island	Post-1200 AD	Long bone
	EI8	Easter Island	Post-1200 AD	Long bone
	Trinitatis	Denmark	18th century	Long bone
Brief pre-digestion	ANR 4704	Easter Island	Post-1200 AD	Cranial bone
	ANR 4705	Easter Island	Post-1200 AD	Cranial bone
	ANR 4706	Easter Island	Post-1200 AD	Cranial bone
	ANR 4707	Easter Island	Post-1200 AD	Cranial bone
	ANR 4708	Easter Island	Post-1200 AD	Cranial bone
	ANR 4709	Easter Island	Post-1200 AD	Cranial bone
	ANR 4710	Easter Island	Post-1200 AD	Cranial bone
	ANR 4712	Easter Island	Post-1200 AD	Cranial bone
	ANR 4714	Easter Island	Post-1200 AD	Cranial bone
	ANR 4715	Easter Island	Post-1200 AD	Cranial bone
	Rise 479	Hungary	Bronze Age, 2000-1500 BC	Long bone
	Rise 483	Hungary	Bronze Age, 2000-1500 BC	Long bone
	AAG1_1	Guadeloupe	400-1400 AD	Long bone
	AAG2_1	Guadeloupe	400-1400 AD	Long bone
	AAG3_1	Guadeloupe	400-1400 AD	Long bone
	AAG4_1	Guadeloupe	400-1400 AD	Long bone
	AAG5_1	Guadeloupe	400-1400 AD	Long bone
	AAG6_1	Guadeloupe	400-1400 AD	Tooth
	AAG7_1	Guadeloupe	400-1400 AD	Tooth
	AAG8_1	Guadeloupe	400-1400 AD	Tooth
	AAG9_1	Guadeloupe	400-1400 AD	Tooth
Cementum vs dentine	Trinitatis 1	Denmark	18th century	Tooth
	Trinitatis 2	Denmark	18th century	Tooth
	ANR 4709	Easter Island	Post-1200 AD	Tooth
	ANR 4711	Easter Island	Post-1200 AD	Tooth
	ANR 4713	Easter Island	Post-1200 AD	Tooth
	VHM00500 X7	Denmark	Iron Age, c.100 AD	Tooth
	VHM00500 X22	Denmark	Iron Age, c.100 AD	Tooth
	VHM00500 X73	Denmark	Iron Age, c.100 AD	Tooth
	VHM00500 X77	Denmark	Iron Age, c.100 AD	Tooth
	VHM00500 X81	Denmark	Iron Age, c.100 AD	Tooth
	ID-530	Greenland	c.1100 AD	Tooth
	ID-532	Greenland	c.1100 AD	Tooth
	ID-677	Greenland	c.1100 AD	Tooth
	ID-678	Greenland	c.1100 AD	Tooth

**Table 2:** Contamination estimates.

Experiment type	Sample	Mitogenome coverage	Estimated contaminated fraction	95 % probability interval
Pre-digestion time	Rise479 A	10.6 X	45.0%^1^	37.5 to 53.7%^1^
	Rise479 B	16.7 X	1.2%	0.2 to 3.9 %
	Rise483 A	14.8 X	2.5%	0.4 to 5.7%
	Rise483 B	5.9 X	0.3%	0.0 to 6.0%
Dentine vs. cementum-enriched	ID-530_A	8.8 X	1.1%	0.1 to 5.3%
	ID-530_B	11.8 X	0.9%	0.1 to 3.5%
	ID-532_A	10.1 X	1.4%	0.2 to 4.5%
	ID-532_B	33.6 X	0.7%	0.2 to 2.3%
	VHM00500 X7_A	15.7 X	0.3%	0.0 to 4.8%
	VHM00500 X7_B	16.7 X	0.2%	0.0 to 3.1%
	VHM00500 X81_A	18.2 X	19.5%	15.3 to 25.5%
	VHM00500 X81_B	21.8 X	0.1%	0.0 to 1.3%

The values represent the estimated fraction of human mtDNA contaminants as determined using contamMix. In the pre-digestion experiment, samples named “B” were pre-digested as opposed to samples named “A”. In the dentine vs. root surface experiments, samples named “B” represent the surface as opposed to the inner dentine named “A”.

^1^estimated using the consensus of Rise479 B as reference.

**Table 3:**
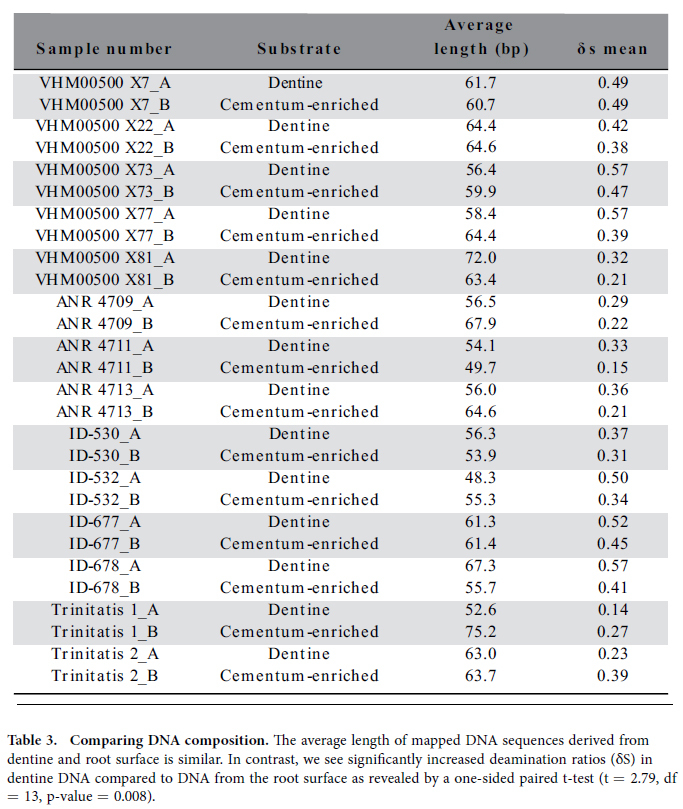
Comparing DNA composition.
