# Supplementary material for: Improving access to endogenous DNA in ancient bones and teeth
Source: Sci Rep. 2015 Jun 17;5:11184. doi: 10.1038/srep11184 (PMC4472031; doi:10.1038/srep11184)
Supplement: Supplementary Information [file srep11184-s1.pdf]

Improving access to endogenous DNA in ancient bones and teeth

Peter B. Damgaard[1], Ashot Margaryan[1], Hannes Schroeder[1,2], Ludovic Orlando[1], Eske Willerslev[1], Morten E. Allentoft[1*]

1) Centre for GeoGenetics, Natural History Museum, University of Copenhagen
2) Faculty of Archaeology, Leiden University, PO Box 9515, 2300 Leiden, The Netherlands
* Corresponding author: morten.allentoft@gmail.com

– Supplementary Material –

Table of Contents

1 IMPROVING ACCESS TO ENDOGENOUS DNA IN ANCIENT BONES AND TEETH........III

1.1 BASIC DNA SEQUENCING STATISTICS........III

1.2 EFFECT OF PRE-DIGESTION ON GC-CONTENT........VII

1.3 EFFECT OF PRE-DIGESTION ON DAMAGE PARAMETERS........XIII

2 SUPPLEMENTARY METHODS S1........XXI

REFERENCES........XXIII

1 Improving access to endogenous DNA in ancient bones and teeth

1.1 Basic DNA sequencing statistics

Sample	Total downsampled sequences	Retained sequences	Mapped sequences to Hg19	Non-duplicate human sequences	Clonality (%)	Endogenous DNA content (%)
EI9A	1.24E+7	1.03E+7	5.34E+2	5.16E+2	3.37E+0	5.17E-3
EI9B	1.24E+7	1.08E+7	7.83E+2	7.26E+2	7.28E+0	7.27E-3
EI9C	1.24E+7	1.07E+7	6.34E+2	6.19E+2	2.37E+0	5.95E-3
EI9D	1.24E+7	1.01E+7	7.35E+2	7.12E+2	3.13E+0	7.30E-3
EI9E	1.24E+7	1.03E+7	7.59E+2	7.34E+2	3.29E+0	7.39E-3
EI9F	1.24E+7	1.20E+7	3.18E+2	3.07E+2	3.46E+0	2.64E-3
EI19A	3.69E+6	3.62E+6	9.90E+1	9.50E+1	4.04E+0	2.73E-3
EI19B	3.69E+6	3.46E+6	7.90E+1	7.80E+1	1.27E+0	2.28E-3
EI19C	3.69E+6	3.52E+6	8.20E+1	8.00E+1	2.44E+0	2.33E-3
EI19D	3.69E+6	3.44E+6	8.20E+1	8.10E+1	1.22E+0	2.38E-3
EI19E	3.69E+6	3.36E+6	8.50E+1	8.50E+1	0.00E+0	2.53E-3
EI19F	3.69E+6	3.61E+6	6.50E+1	6.40E+1	1.54E+0	1.80E-3
EI22A	1.26E+7	1.16E+7	1.80E+3	1.78E+3	1.44E+0	1.55E-2
EI22B	1.26E+7	1.15E+7	2.38E+3	2.35E+3	1.22E+0	2.06E-2
EI22C	1.26E+7	1.16E+7	2.21E+3	2.18E+3	1.13E+0	1.90E-2
EI22D	1.26E+7	1.15E+7	2.20E+3	2.16E+3	1.91E+0	1.92E-2
EI22E	1.26E+7	1.17E+7	2.55E+3	2.51E+3	1.30E+0	2.17E-2
EI22F	1.26E+7	1.24E+7	6.50E+2	6.46E+2	6.15E-1	5.26E-3
EI8A	1.38E+7	1.36E+7	1.32E+5	1.29E+5	2.33E+0	9.70E-1
EI8B	1.38E+7	1.36E+7	8.43E+4	8.31E+4	1.45E+0	6.20E-1
EI8C	1.38E+7	1.36E+7	1.41E+5	1.32E+5	6.74E+0	1.04E+0
EI8D	1.38E+7	1.37E+7	2.00E+5	1.79E+5	1.04E+1	1.47E+0
EI8E	1.38E+7	1.36E+7	2.26E+5	1.84E+5	1.85E+1	1.66E+0
EI8F	1.38E+7	1.36E+7	1.68E+5	1.66E+5	1.37E+0	1.24E+0
TrinitatisA	3.35E+6	3.17E+6	2.78E+3	2.77E+3	2.16E-1	8.76E-2
TrinitatisB	3.35E+6	2.94E+6	2.17E+3	2.15E+3	6.92E-1	7.38E-2
TrinitatisC	3.35E+6	3.15E+6	1.46E+3	1.45E+3	6.86E-1	4.62E-2
TrinitatisD	3.35E+6	3.01E+6	1.49E+3	1.49E+3	2.01E-1	4.96E-2
TrinitatisE	3.35E+6	3.20E+6	1.60E+3	1.59E+3	6.27E-1	4.98E-2
TrinitatisF	3.35E+6	3.17E+6	4.40E+2	4.38E+2	4.55E-1	1.39E-2

Supplementary Table S1: Basic mapping statistics for the five bones used in the pre-digestion length experiment (ie. sample F: no pre-digestion, sample A-E: respectively 30 min, 1 hour, 2 hours, 3 hours, 6 hours of pre-digestion). For comparative purposes the six sequencing files from each sample were randomly donwsampled to contain the same number of raw DNA sequences. The endogenous content represents the fraction of sequences that pass trimming that mapped to Hg19. The clonality of the library is estimated as the fraction of the human sequences that are clonal.

Sample	Total downsampled sequences	Retained sequences	Mapped sequences to Hg19	Non-duplicate human sequences	Clonality (%)	Endogenous DNA content (%)
ANR 4704_A	9.44E+6	9.09E+6	2.33E+2	2.31E+2	8.58E-1	2.56E-3
ANR 4704_B	9.44E+6	8.80E+6	3.98E+2	3.93E+2	1.26E+0	4.52E-3
ANR 4705_A	2.13E+7	1.98E+7	3.08E+3	3.04E+3	1.23E+0	1.55E-2
ANR 4705_B	2.13E+7	1.91E+7	4.43E+3	4.37E+3	1.33E+0	2.32E-2
ANR 4706_A	1.38E+7	1.20E+7	1.53E+4	1.53E+4	2.74E-1	1.28E-1
ANR 4706_B	1.38E+7	1.14E+7	1.16E+4	1.15E+4	8.20E-1	1.02E-1
ANR 4707_A	1.44E+7	1.35E+7	1.09E+5	1.07E+5	1.89E+0	8.02E-1
ANR 4707_B	1.44E+7	1.29E+7	1.06E+5	1.03E+5	2.60E+0	8.19E-1
ANR 4708_A	1.29E+7	1.18E+7	3.15E+4	3.13E+4	4.70E-1	2.67E-1
ANR 4708_B	1.29E+7	1.24E+7	3.24E+4	3.17E+4	2.16E+0	2.61E-1
ANR 4709_A	1.84E+7	1.34E+7	5.87E+4	5.54E+4	5.59E+0	4.39E-1
ANR 4709_B	1.84E+7	1.18E+7	1.78E+5	1.76E+5	1.47E+0	1.51E+0
ANR 4710_A	1.59E+7	1.09E+7	1.68E+3	1.63E+3	2.91E+0	1.55E-2
ANR 4710_B	1.59E+7	1.01E+7	1.11E+3	1.10E+3	1.08E+0	1.10E-2
ANR 4711_A	1.31E+7	7.10E+6	1.62E+4	1.52E+4	6.56E+0	2.29E-1
ANR 4712_B	1.31E+7	7.65E+6	6.42E+3	6.30E+3	1.82E+0	8.39E-2
ANR 4714_A	8.92E+6	6.80E+6	1.23E+4	1.17E+4	4.49E+0	1.80E-1
ANR 4714_B	8.92E+6	7.02E+6	3.57E+4	3.29E+4	7.97E+0	5.09E-1
ANR 4715_A	1.61E+7	9.94E+6	3.76E+3	3.61E+3	3.91E+0	3.78E-2
ANR 4715_B	1.61E+7	1.00E+7	3.37E+3	3.25E+3	3.62E+0	3.37E-2
AAG1_A	2.90E+6	2.69E+6	3.64E+2	3.64E+2	0.00E+0	1.36E-2
AAG1_B	2.90E+6	2.60E+6	5.62E+2	5.60E+2	3.56E-1	2.16E-2
AAG2_A	3.17E+6	2.90E+6	8.66E+2	8.66E+2	0.00E+0	2.99E-2
AAG2_B	3.17E+6	2.85E+6	2.07E+3	2.05E+3	6.29E-1	7.26E-2
AAG3_A	1.61E+6	1.50E+6	7.00E+1	7.00E+1	0.00E+0	4.68E-3
AAG3_B	1.61E+6	1.52E+6	1.07E+2	1.07E+2	0.00E+0	7.06E-3
AAG4_A	3.21E+6	3.00E+6	5.16E+2	5.15E+2	1.94E-1	1.72E-2
AAG4_B	3.21E+6	2.85E+6	9.67E+2	9.64E+2	3.10E-1	3.39E-2
AAG5_A	3.42E+6	3.21E+6	7.95E+2	7.95E+2	0.00E+0	2.47E-2
AAG5_B	3.42E+6	3.09E+6	1.55E+3	1.53E+3	8.40E-1	5.00E-2
AAG6_A	2.34E+6	2.12E+6	5.43E+3	5.43E+3	1.29E-1	2.57E-1
AAG6_B	2.34E+6	2.06E+6	1.90E+4	1.89E+4	2.74E-1	9.21E-1
AAG7_A	2.71E+6	2.47E+6	1.81E+4	1.80E+4	1.77E-1	7.32E-1
AAG7_B	2.71E+6	2.41E+6	5.43E+4	5.41E+4	4.48E-1	2.25E+0
AAG8_A	3.43E+6	2.95E+6	4.58E+4	4.57E+4	2.92E-1	1.55E+0
AAG8_B	3.43E+6	3.01E+6	1.33E+4	1.33E+4	2.10E-1	4.42E-1
AAG9_A	2.61E+6	2.40E+6	8.77E+3	8.76E+3	6.84E-2	3.66E-1
AAG9_B	2.61E+6	2.32E+6	2.67E+4	2.66E+4	3.11E-1	1.15E+0
Rise479_A	1.41E+7	1.30E+7	5.01E+5	4.78E+5	4.65E+0	3.85E+0
Rise479_B	1.41E+7	1.25E+7	8.60E+5	7.64E+5	1.12E+1	6.88E+0
Rise483_A	1.75E+7	1.68E+7	1.69E+5	1.66E+5	1.81E+0	1.01E+0
Rise483_B	1.75E+7	1.59E+7	2.33E+5	1.40E+5	3.97E+1	1.47E+0

Supplementary Table S2: Basic mapping statistics for the bones used in the short pre-digestions experiments (15-30 mins). Samples B (gray) represent pre-digested samples. For comparative purposes the two sequencing files from each pair were randomly donwsampled to contain the same number of raw DNA sequences. The endogenous content represents the fraction of sequences that pass trimming that mapped to Hg19. The clonality of the library is estimated as the fraction of the human sequences that are clonal.

Sample	Total downsampled sequences	Retained sequences	Mapped sequences to Hg19	Non-duplicate human sequences	Clonality (%)	Endogenous DNA content (%)
ANR 4709_A	9.05E+6	8.60E+6	5.10E+4	5.08E+4	4.55E-1	5.93E-1
ANR 4709_B	9.05E+6	8.73E+6	2.93E+5	2.91E+5	5.00E-1	3.35E+0
ANR 4711_A	9.21E+6	8.89E+6	2.07E+4	2.07E+4	2.65E-1	2.33E-1
ANR 4711_B	9.21E+6	8.78E+6	9.43E+4	9.39E+4	4.37E-1	1.07E+0
ANR 4713_A	1.00E+7	9.54E+6	3.85E+4	3.83E+4	3.25E-1	4.03E-1
ANR 4713_B	1.00E+7	9.62E+6	2.65E+4	2.65E+4	2.11E-1	2.76E-1
VHM00500 X7_A	7.62E+6	7.28E+6	1.57E+4	1.57E+4	1.21E-1	2.16E-1
VHM00500 X7_B	7.62E+6	7.02E+6	1.17E+5	1.17E+5	2.85E-1	1.67E+0
VHM00500 X22_A	7.20E+6	6.65E+6	3.96E+5	3.90E+5	1.42E+0	5.95E+0
VHM00500 X22_B	7.20E+6	6.91E+6	1.97E+6	1.95E+6	1.04E+0	2.85E+1
VHM00500 X73_A	4.24E+6	3.71E+6	1.03E+4	1.02E+4	8.53E-1	2.78E-1
VHM00500 X73_B	4.24E+6	3.83E+6	1.49E+5	1.48E+5	4.71E-1	3.90E+0
VHM00500 X77_A	6.78E+6	5.92E+6	7.76E+3	7.74E+3	2.96E-1	1.31E-1
VHM00500 X77_B	6.78E+6	5.80E+6	4.52E+4	4.51E+4	3.03E-1	7.80E-1
VHM00500 X81_A	7.79E+6	6.88E+6	7.27E+4	7.19E+4	1.10E+0	1.06E+0
VHM00500 X81_B	7.79E+6	7.15E+6	6.71E+5	6.64E+5	1.02E+0	9.38E+0
ID-530_A	1.76E+7	1.73E+7	1.27E+6	1.25E+6	1.87E+0	7.37E+0
ID-530_B	1.76E+7	1.72E+7	1.13E+7	1.11E+7	1.13E+0	6.54E+1
ID-532_A	1.41E+7	1.11E+7	1.94E+6	1.88E+6	3.27E+0	1.75E+1
ID-532_B	1.41E+7	1.33E+7	5.96E+6	5.81E+6	2.43E+0	4.48E+1
ID-677_A	1.77E+6	1.72E+6	1.56E+4	1.55E+4	7.37E-1	9.07E-1
ID-677_B	1.77E+6	1.73E+6	2.11E+4	2.11E+4	1.61E-1	1.22E+0
ID-678_A	2.42E+7	2.37E+7	1.51E+5	1.50E+5	8.34E-1	6.39E-1
ID-678_B	2.42E+7	2.22E+7	1.03E+5	1.02E+5	2.77E-1	4.63E-1
Trinitatis 1_A	6.98E+6	6.62E+6	4.03E+5	4.00E+5	8.50E-1	6.09E+0
Trinitatis 1_B	6.98E+6	6.88E+6	1.44E+5	1.42E+5	9.86E-1	2.09E+0
Trinitatis 2_A	7.17E+6	7.04E+6	4.77E+5	4.71E+5	1.22E+0	6.77E+0
Trinitatis 2_B	7.17E+6	6.88E+6	1.09E+6	1.08E+6	9.60E-1	1.59E+1

Supplementary Table S3: Basic mapping statistics for the dentine/cementum-enriched fraction comparison. Samples A represent dentine samples while samples B (gray) represent cementum-enriched samples. For comparative purposes the two sequencing files from each pair were randomly donwsampled to contain the same number of raw DNA sequences. The endogenous content represents the fraction of sequences that pass trimming that mapped to Hg19. The clonality of the library is estimated as the fraction of the human sequences that are clonal.

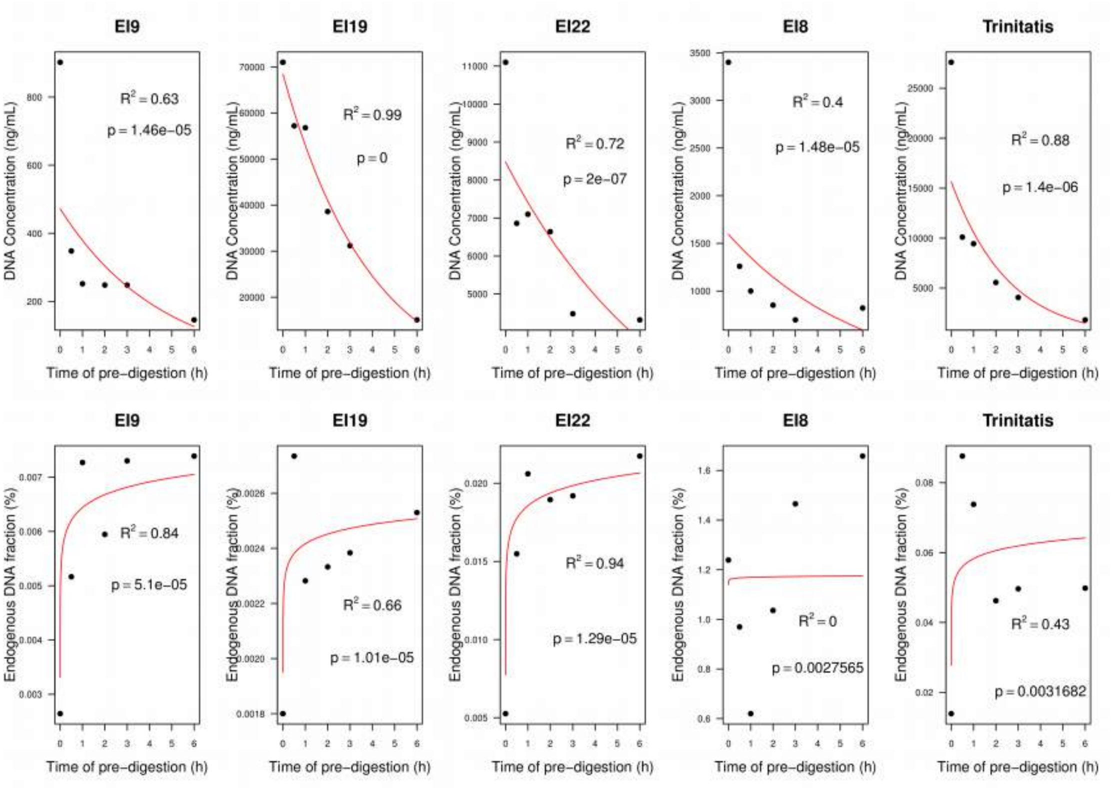

Supplementary Figure S1: Top row (A-E) shows DNA concentrations as measured by Qubit® Fluorometric Quantitation (Life Technologies, Grand Island, NY) as a function of pre-digestion time. An exponential model was fitted to the data (red line) and the respective R^2 values (rounded to two 2 decimals) and p-values (rounded to 7 decimals) were printed. Second row (F-J) shows the endogenous DNA content as a function of pre-digestion time. A logarithmic model was fitted to the data (red line) and the respective R^2 values (rounded to two 2 decimals) and p-values (rounded to 7 decimals) were printed.

1.2 Effect of pre-digestion on GC-content

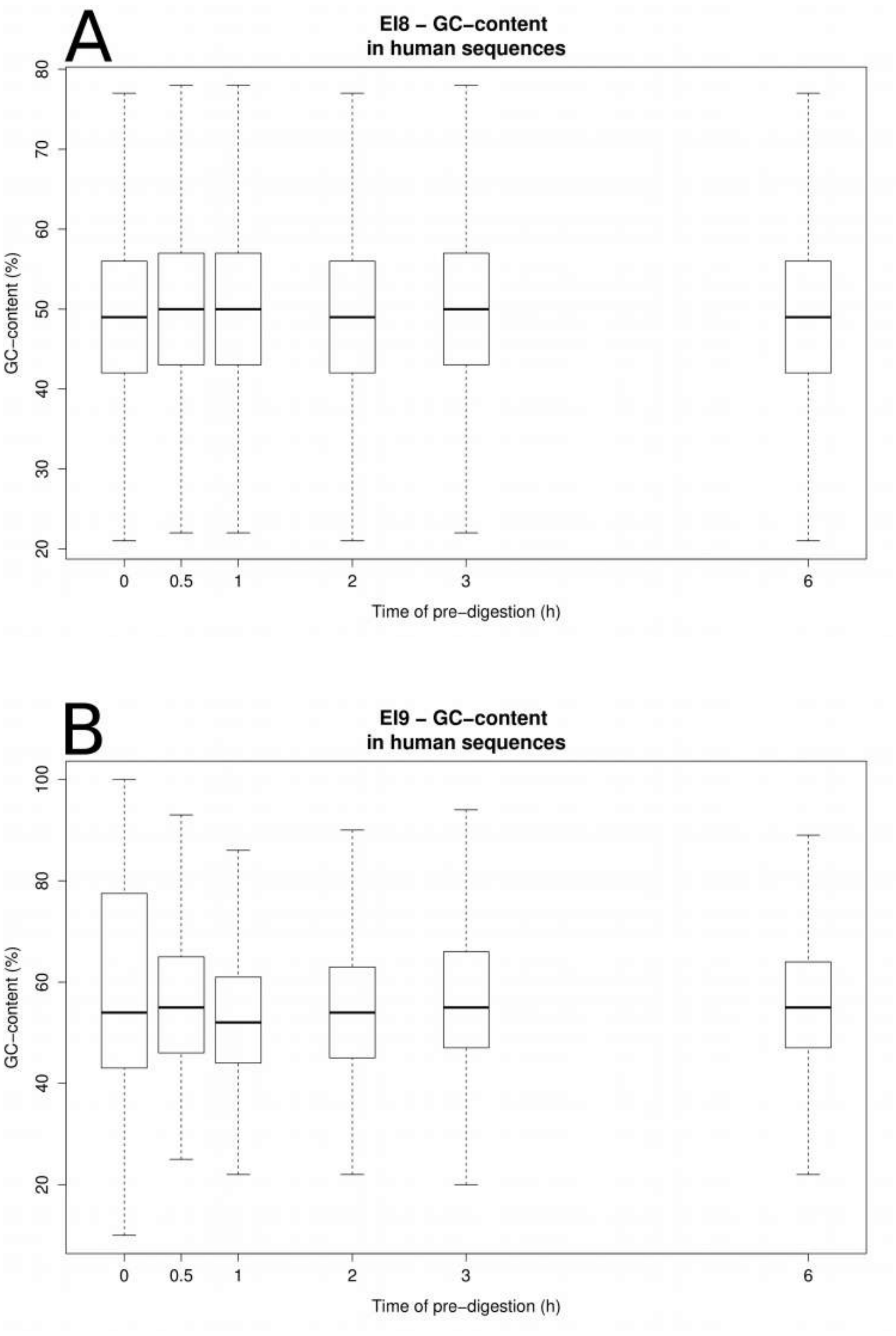

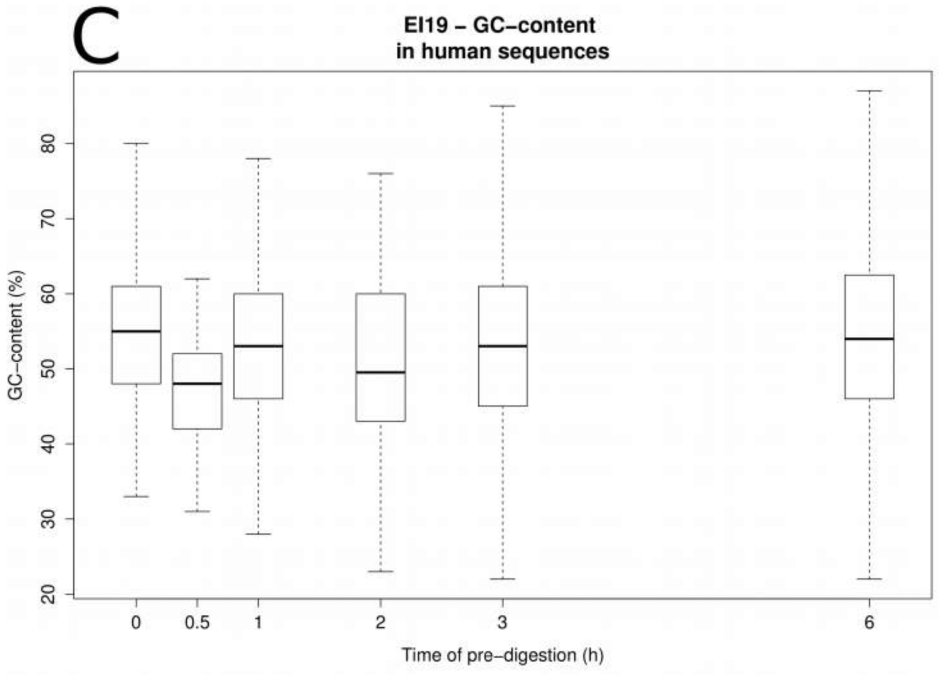
C
EI19 - GC-content
in human sequences
GC-content (%)
20
30
40
50
60
70
80
0
0.5
1
2
3
6
Time of pre-digestion (h)

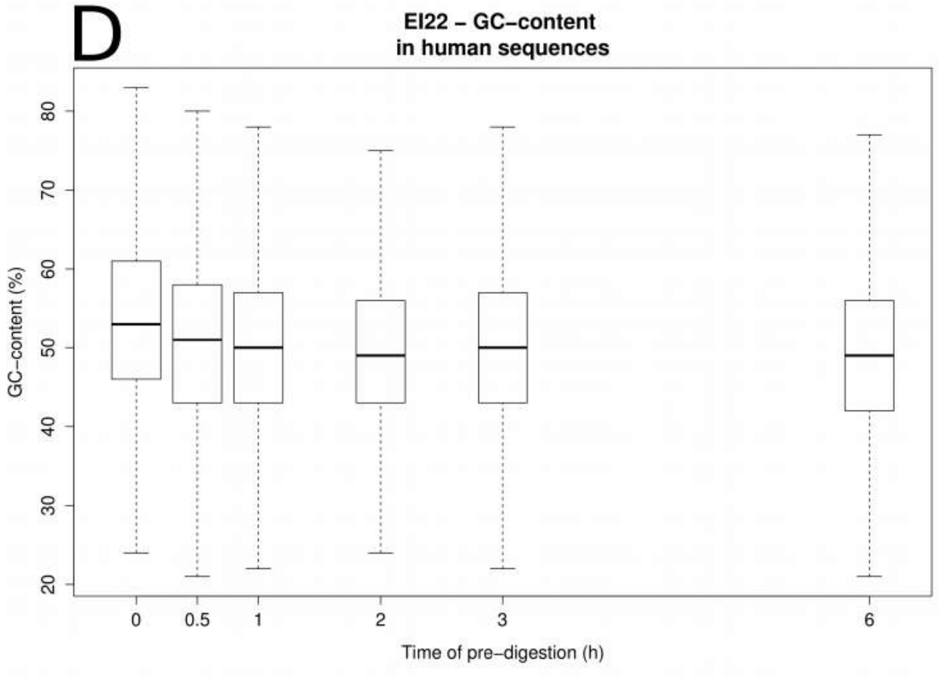
D
EI22 - GC-content
in human sequences
GC-content (%)
20
30
40
50
60
70
80
0
0.5
1
2
3
6
Time of pre-digestion (h)

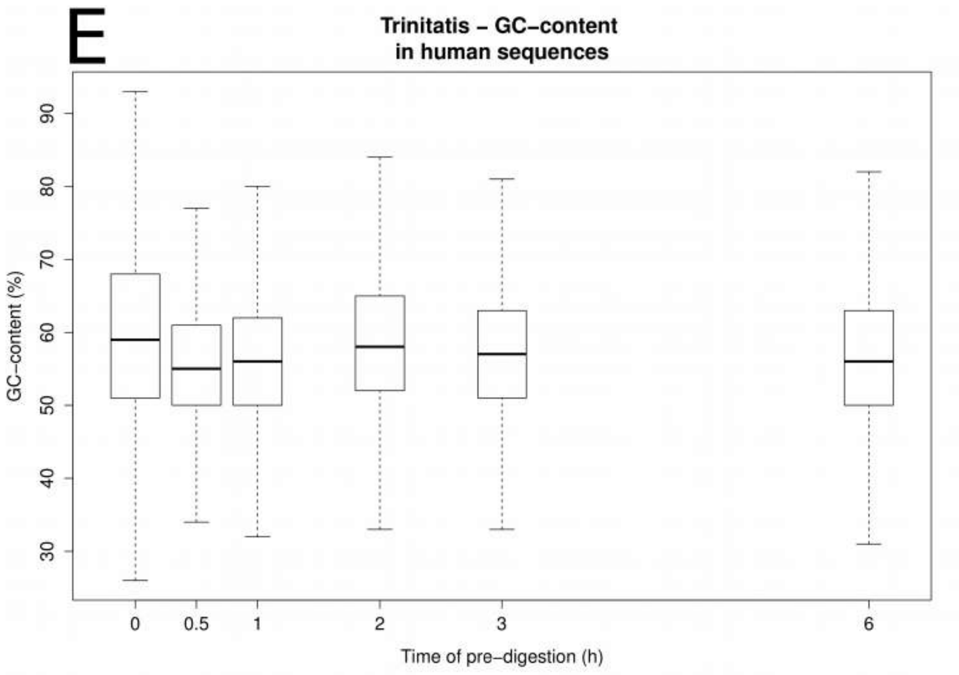

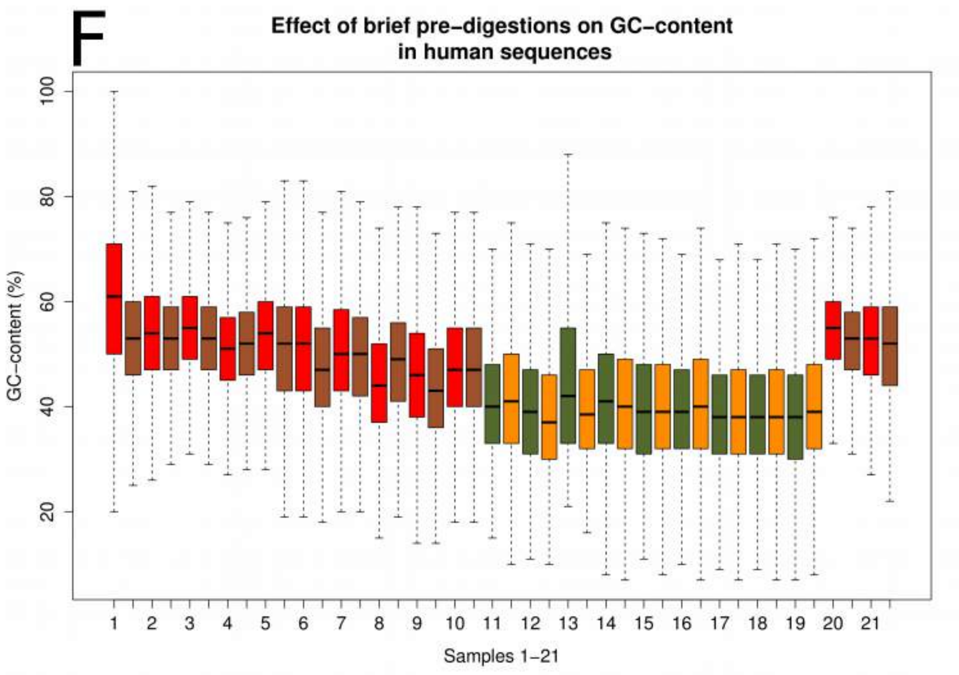

Supplementary Figure S2: A-E: The genomic GC content as a function of pre-digestion time in the DNA sequences identified as human from **(A)** bone EI8, **(B)** bone EI9, **(C)** bone EI19, **(D)** bone EI22, **(E)** bone Trinitatis. Each boxplot displays the median value (black stripe), first and third quartile (length of box is the interquartile range), and the IQR is extended 1.5 times with the whiskers. In **(F)** the genomic GC-content in the identified human sequences in both non-pre-digested (red and green) and briefly pre-digested (15-30 min) (brown and yellow) is similarly plotted. Samples in red and brown were amplified with AmpliTaq Gold DNA Polymerase (Applied Biosystems). Samples in green and yellow were amplified with KAPA HiFi HotStart Uracil+ ReadyMix (KAPA Biosystems, Woburn, MA, USA). Samples 1-21 refer to 1) ANR 4704, 2) ANR 4705, 3) ANR 4706, 4) ANR 4707, 5) ANR 4708, 6) ANR 4709, 7) ANR 4710, 8) ANR 4712, 9) ANR 4714, 10) ANR 4715, 11) AAG1, 12) AAG2, 13) AAG3, 14) AAG4, 15) AAG5, 16) AAG6, 17) AAG7, 18) AAG8, 19) AAG9, 20) Rise 479, 21)Rise 483

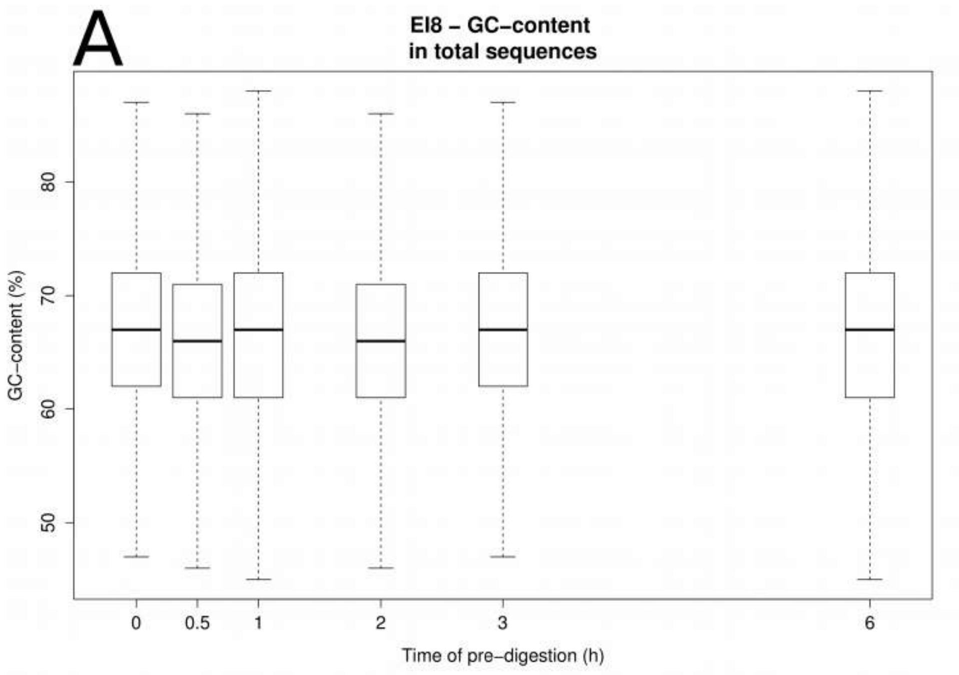
A
EI8 – GC–content
in total sequences
GC–content (%)
50
60
70
80
0
0.5
1
2
3
6
Time of pre–digestion (h)

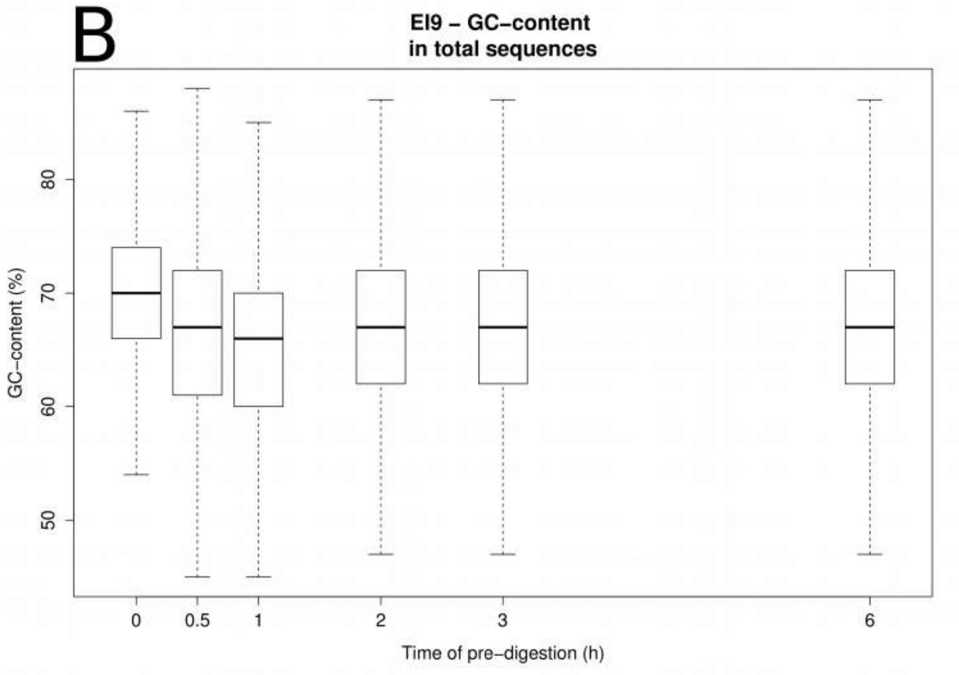
B
EI9 – GC–content
in total sequences
GC–content (%)
50
60
70
80
0
0.5
1
2
3
6
Time of pre–digestion (h)

C

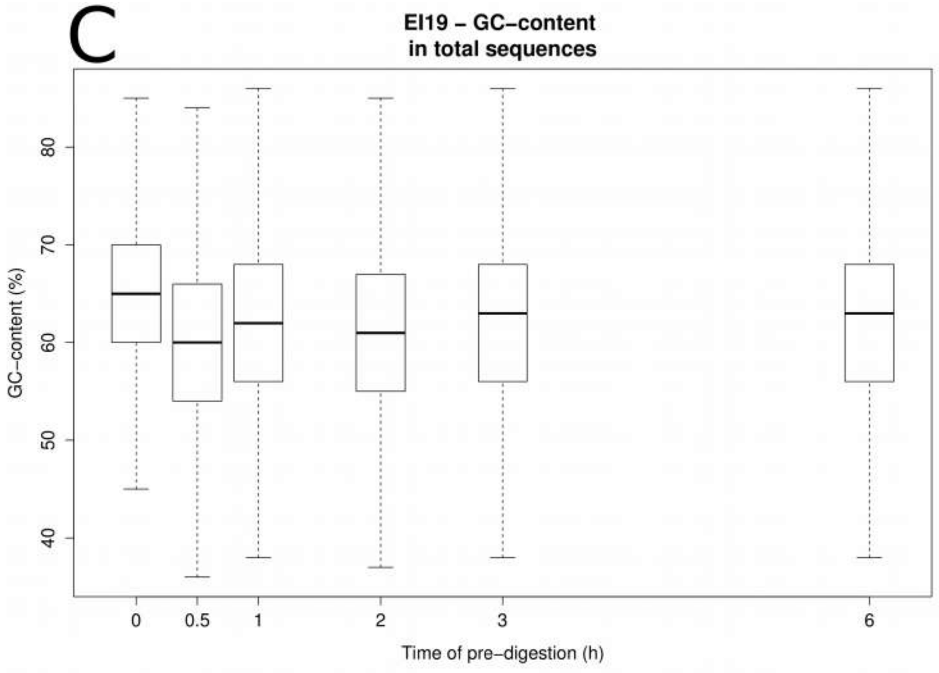
EI19 - GC-content
in total sequences
GC-content (%)
40
50
60
70
80
0
0.5
1
2
3
6
Time of pre-digestion (h)

D

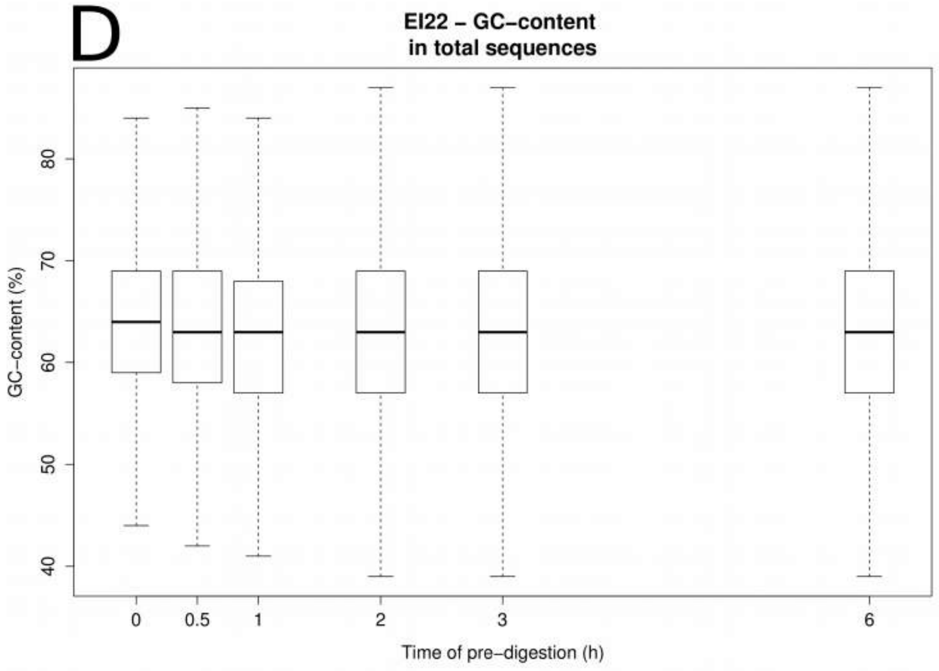
EI22 - GC-content
in total sequences
GC-content (%)
40
50
60
70
80
0
0.5
1
2
3
6
Time of pre-digestion (h)

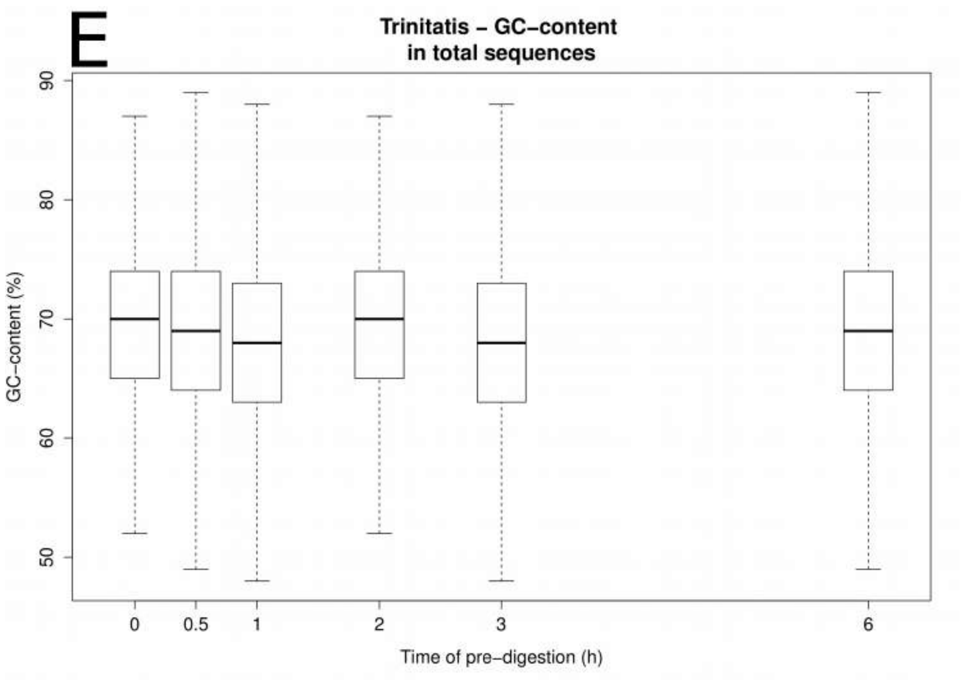

Supplementary Figure S3: A-E: The genomic GC-content in the total (human+non-human) DNA sequences content as a function of pre-digestion time from **(A)** bone EI8, **(B)** bone EI9, **(C)** bone EI19, **(D)** bone EI22, **(E)** bone Trinitatis. Each boxplot displays the median value (black stripe), first and third quartile (length of box is the interquartile range), and the IQR is extended 1.5 times with the whiskers.

1.3 Effect of pre-digestion on damage parameters

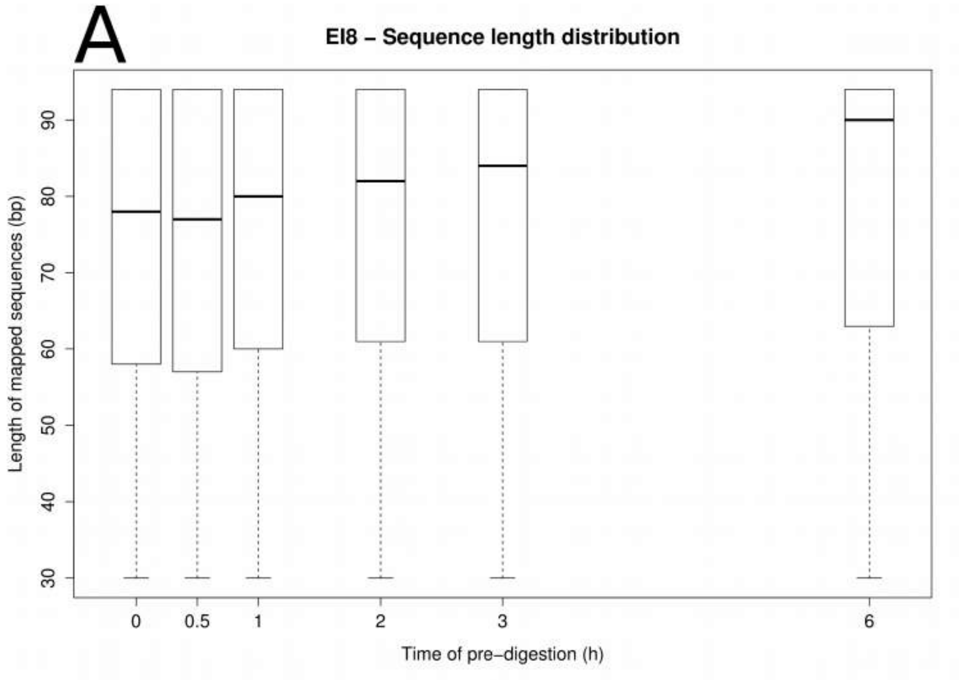

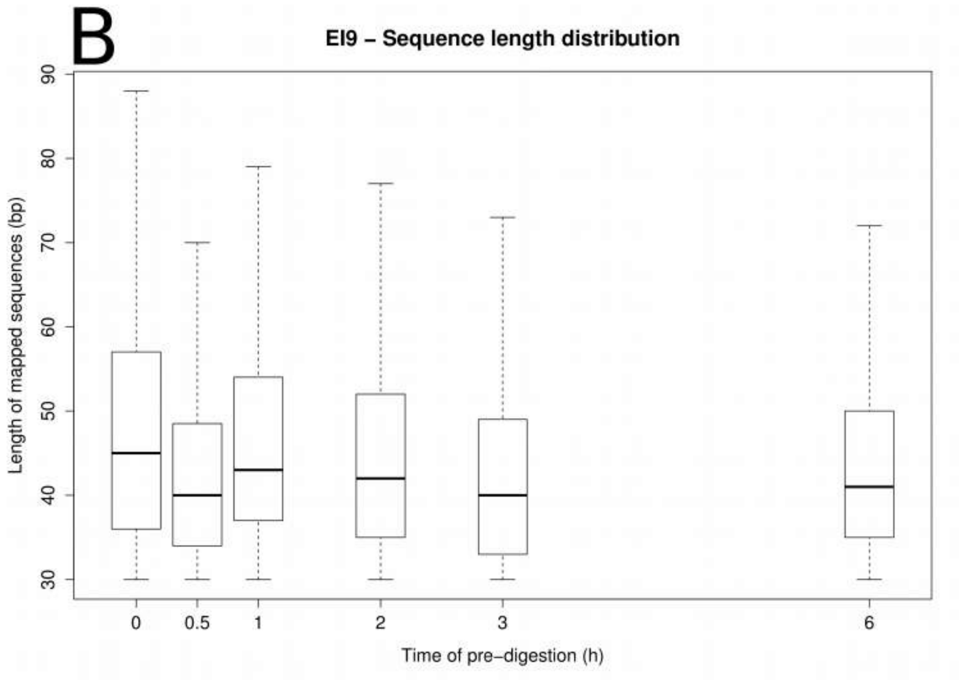

C

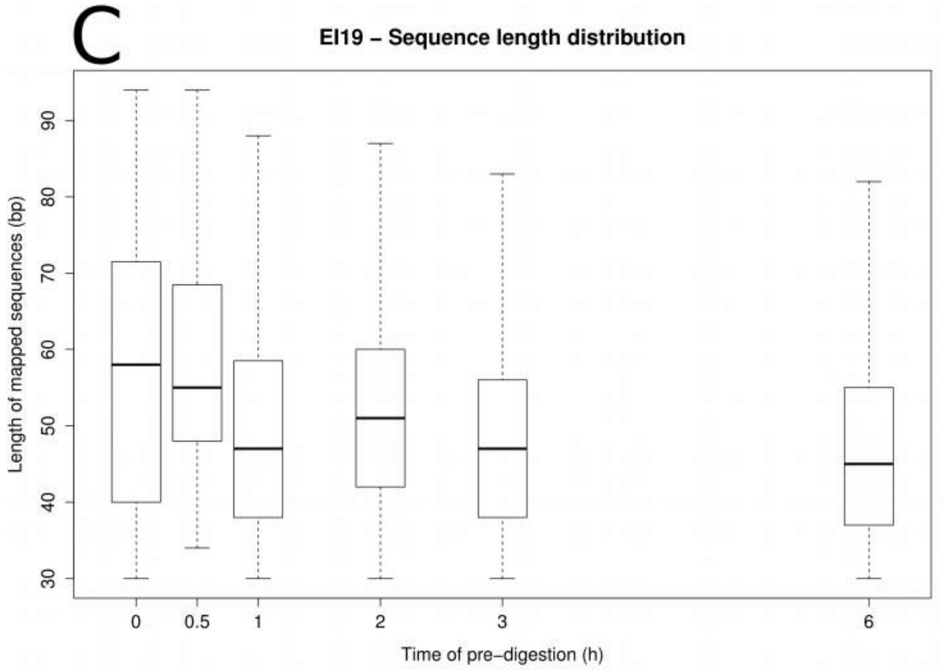
EI19 – Sequence length distribution
Length of mapped sequences (bp)
30
40
50
60
70
80
90
0
0.5
1
2
3
6
Time of pre–digestion (h)

D

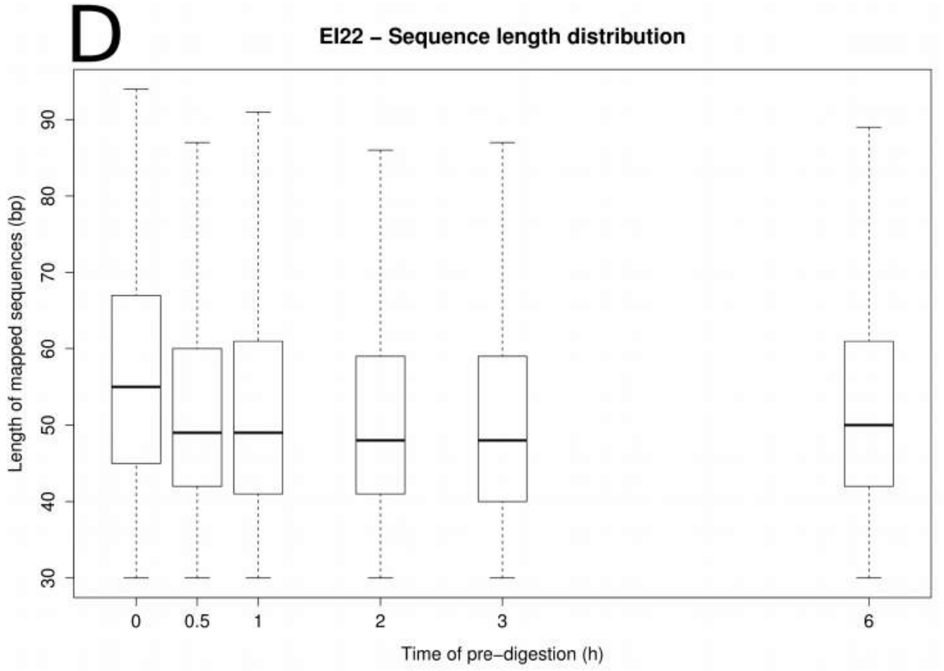
EI22 – Sequence length distribution
Length of mapped sequences (bp)
30
40
50
60
70
80
90
0
0.5
1
2
3
6
Time of pre–digestion (h)

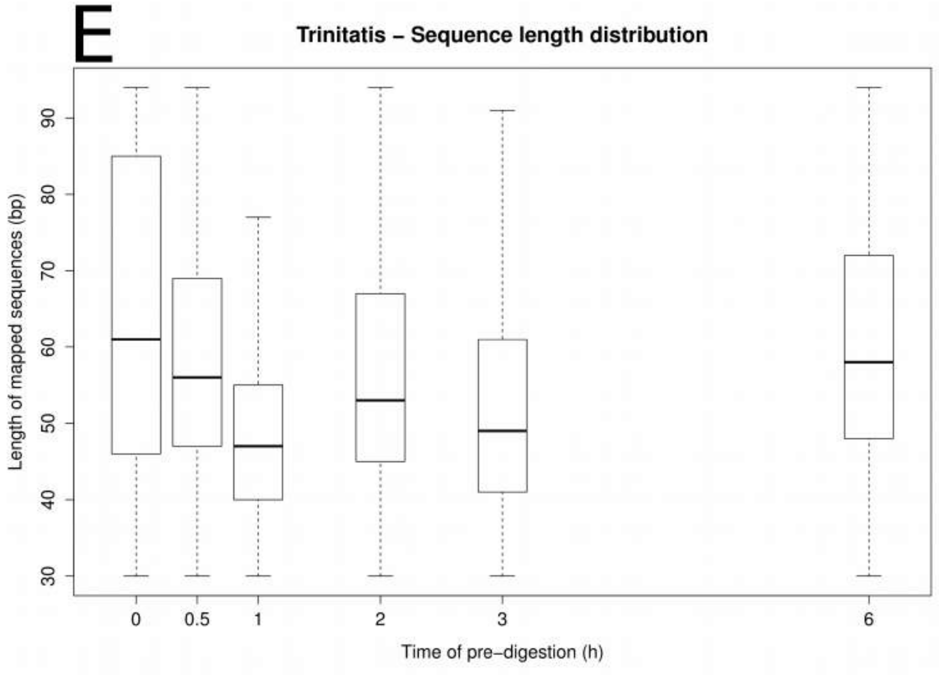

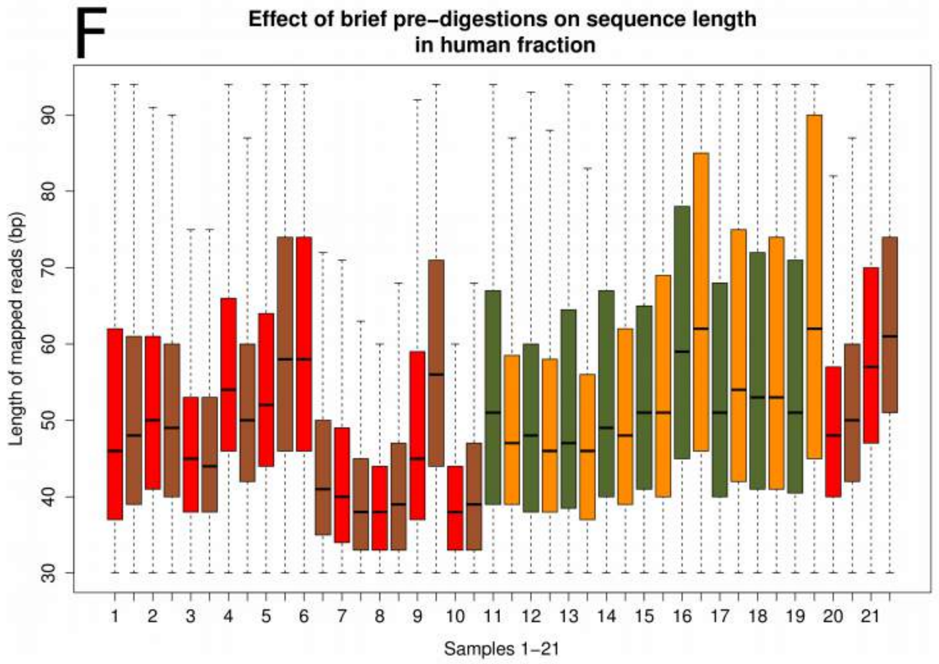

Supplementary Figure S4: A:E DNA sequence length distribution plotted as a function of pre-digestion time for the DNA sequences identified as human from **(A)** bone EI8, **(B)** bone EI9, **(C)** bone EI19, **(D)** bone EI22, **(E)** bone Trinitatis. Each boxplot displays the median value (black stripe), first and third quartile (length of box is the interquartile range), and the IQR is extended 1.5 times with the whiskers. In **(F)** the genomic GC-content in the identified human sequences in both non-pre-digested (red and green) and briefly pre-digested (15-30 min) (brown and yellow) is similarly plotted. Samples in red and brown were amplified with AmpliTaq Gold DNA Polymerase (Applied Biosystems). Samples in green and yellow were amplified with KAPA HiFi HotStart Uracil+ ReadyMix (KAPA Biosystems, Woburn, MA, USA). Samples 1-21 refer to 1) ANR 4704, 2) ANR 4705, 3) ANR 4706, 4) ANR 4707, 5) ANR 4708, 6) ANR 4709, 7) ANR 4710, 8) ANR 4712, 9) ANR 4714, 10) ANR 4715, 11) AAG1, 12) AAG2, 13) AAG3, 14) AAG4, 15) AAG5, 16) AAG6, 17) AAG7, 18) AAG8, 19) AAG9, 20) Rise 479, 21)Rise 483

	Decay constant (k) for non-pre-digested (A)	Decay constant (k) for pre-digested (B)	Average length (bp) of total DNA fragments in non-pre-digested (A)	Average length (bp) of total DNA fragments in pre-digested (B)
ANR 4705	0.06	0.06	16.7	16.7
ANR 4706	0.09	0.09	11.1	11.1
ANR 4707	0.06	0.07	16.7	14.3
ANR 4708	0.06	0.03	16.7	33.3
ANR 4709	0.03	0.08	33.3	12.5
ANR 4710	0.08	0.09	12.5	11.1
ANR 4712	0.1	0.09	10.0	11.1
ANR 4714	0.05	0.03	20.0	33.3
ANR 4715	0.09	0.08	11.1	12.5
AAG2	0.05	0.05	20.0	20.0
AAG4	0.05	0.05	20.0	20.0
AAG5	0.05	0.04	20.0	25.0
AAG6	0.03	0.02	33.3	50.0
AAG7	0.04	0.03	25.0	33.3
AAG8	0.03	0.03	33.3	33.3
AAG9	0.04	0.02	25.0	50.0
Rise 479	0.07	0.07	14.3	14.3
Rise 483	0.04	0.04	25.0	25.0

Supplementary Table S4: The decay constant k (per site) based on the DNA sequence length distributions for each sample calculated as in[1,2] for samples with sufficient sequences. The decay constant k represents the proportion of broken phoshodiester bonds in the DNA strand and is hence an estimator of the degree of DNA fragmentation. The estimated average human DNA fragment length is calculated for each sample as the mean of the fitted exponential distribution: 1/k. Values for pre-digested samples are highlighted in gray.

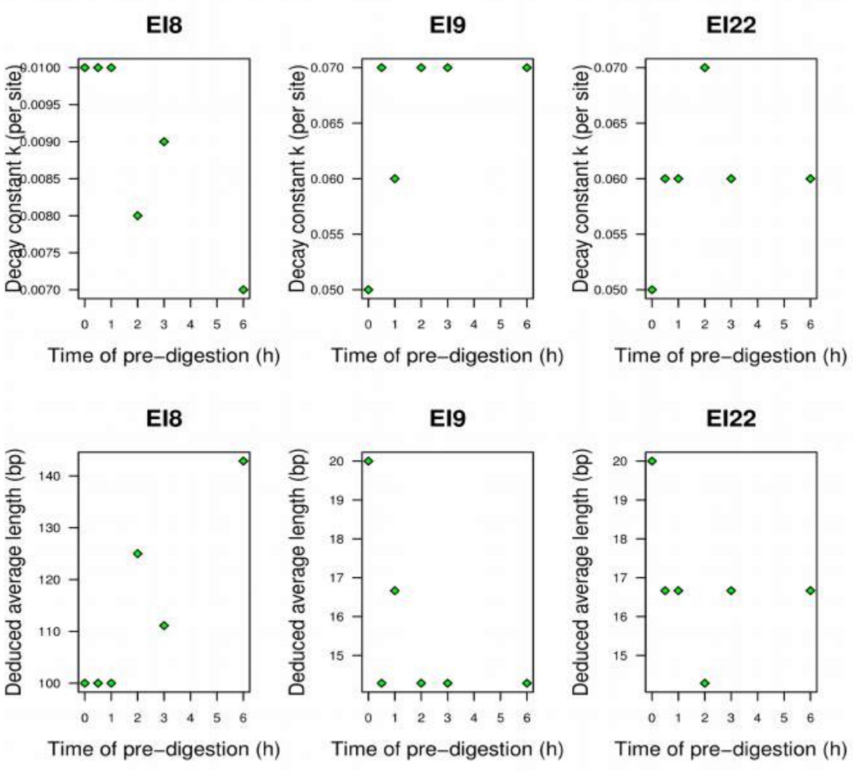

Supplementary Figure S5: Top row: The decay constant k (per site) based on the DNA sequence length distributions for each sample calculated as in[1,2] as a function of pre-digestion time for samples with sufficient sequences. **Bottom row:** The deduced average length of total human DNA fragments as is calculated for each sample as the mean of the fitted exponential distribution: 1/k.

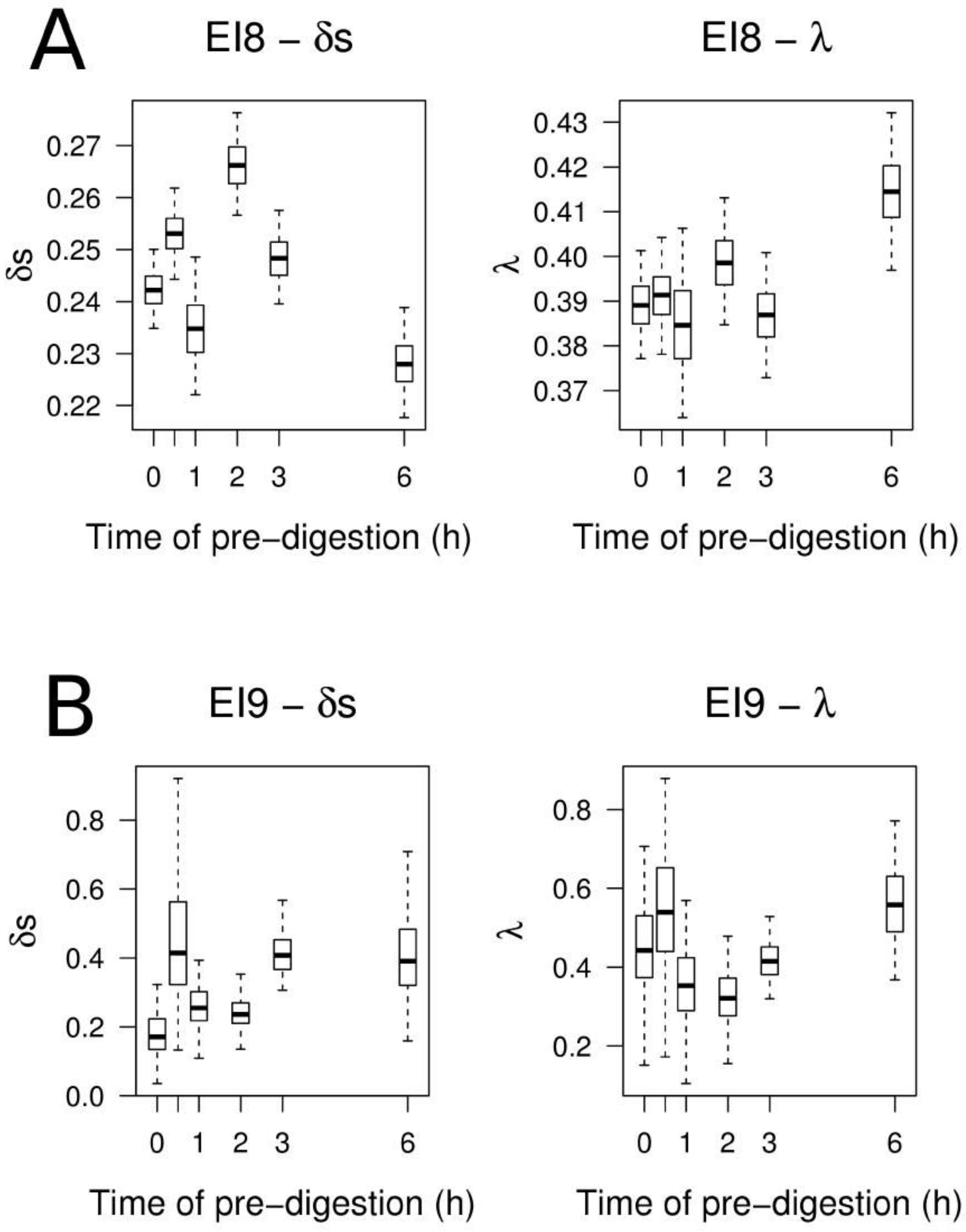
A
EI8 – δs
δs
0.27
0.26
0.25
0.24
0.23
0.22
0 1 2 3 6
Time of pre–digestion (h)
EI8 – λ
λ
0.43
0.42
0.41
0.40
0.39
0.38
0.37
0 1 2 3 6
Time of pre–digestion (h)
B
EI9 – δs
δs
0.8
0.6
0.4
0.2
0.0
0 1 2 3 6
Time of pre–digestion (h)
EI9 – λ
λ
0.8
0.6
0.4
0.2
0 1 2 3 6
Time of pre–digestion (h)

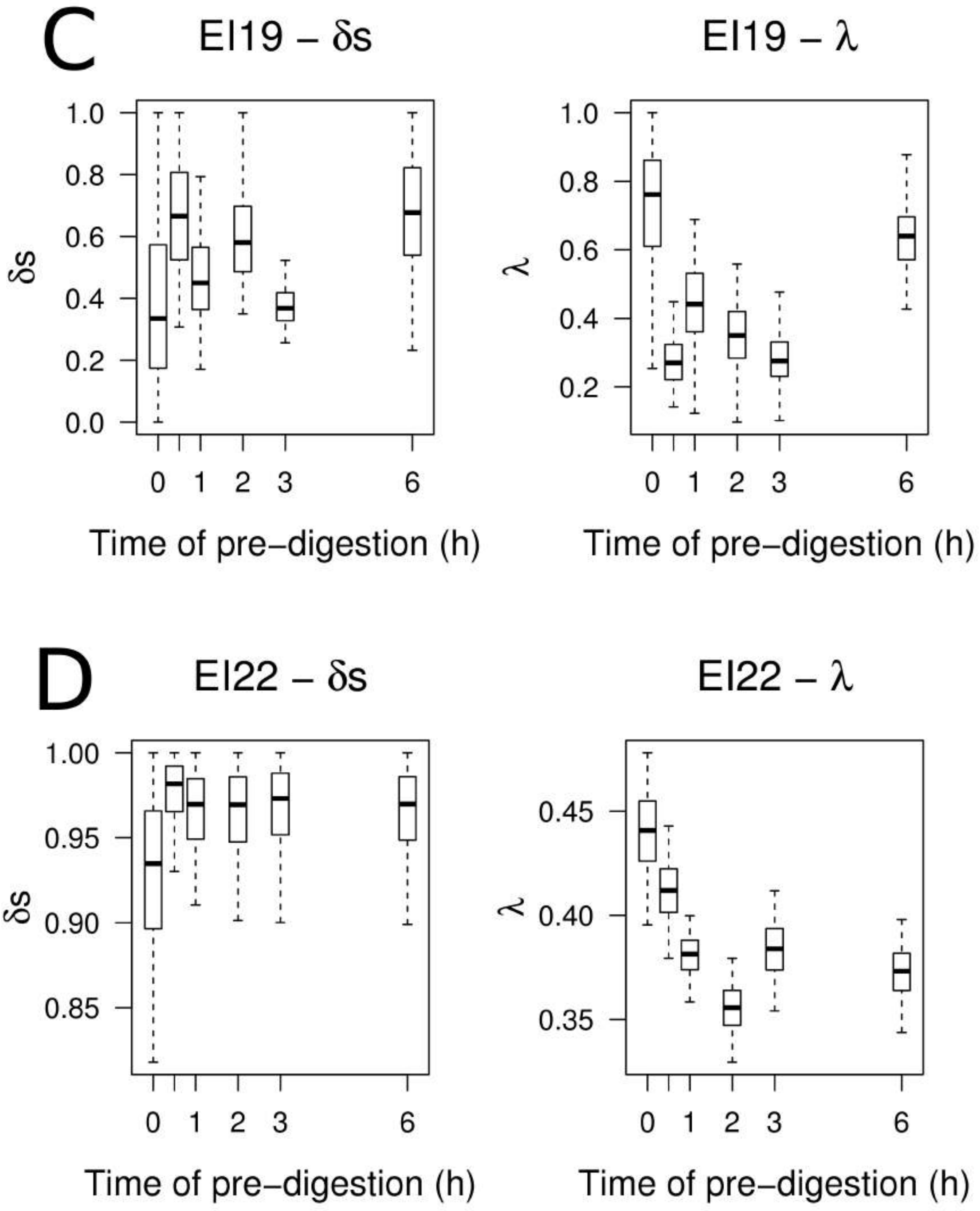
C
EI19 – δs
δs
1.0
0.8
0.6
0.4
0.2
0.0
0 1 2 3 6
Time of pre−digestion (h)
EI19 – λ
λ
1.0
0.8
0.6
0.4
0.2
0 1 2 3 6
Time of pre−digestion (h)
D
EI22 – δs
δs
1.00
0.95
0.90
0.85
0 1 2 3 6
Time of pre−digestion (h)
EI22 – λ
λ
0.45
0.40
0.35
0 1 2 3 6
Time of pre−digestion (h)

E

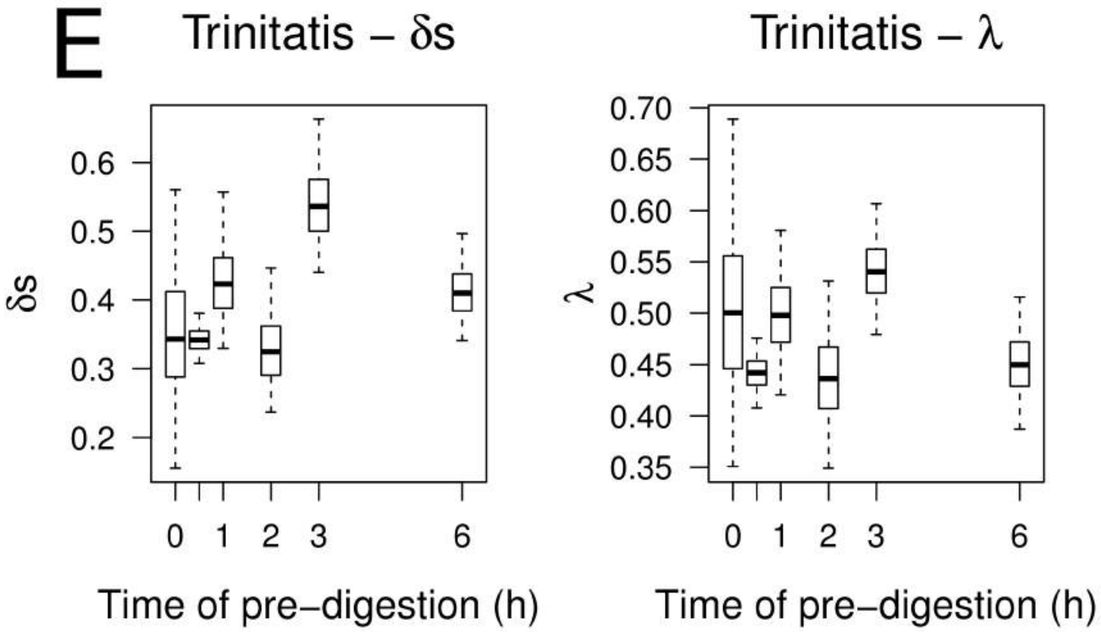
Trinitatis – δs
Trinitatis – λ
δs
λ
Time of pre−digestion (h)
Time of pre−digestion (h)

F

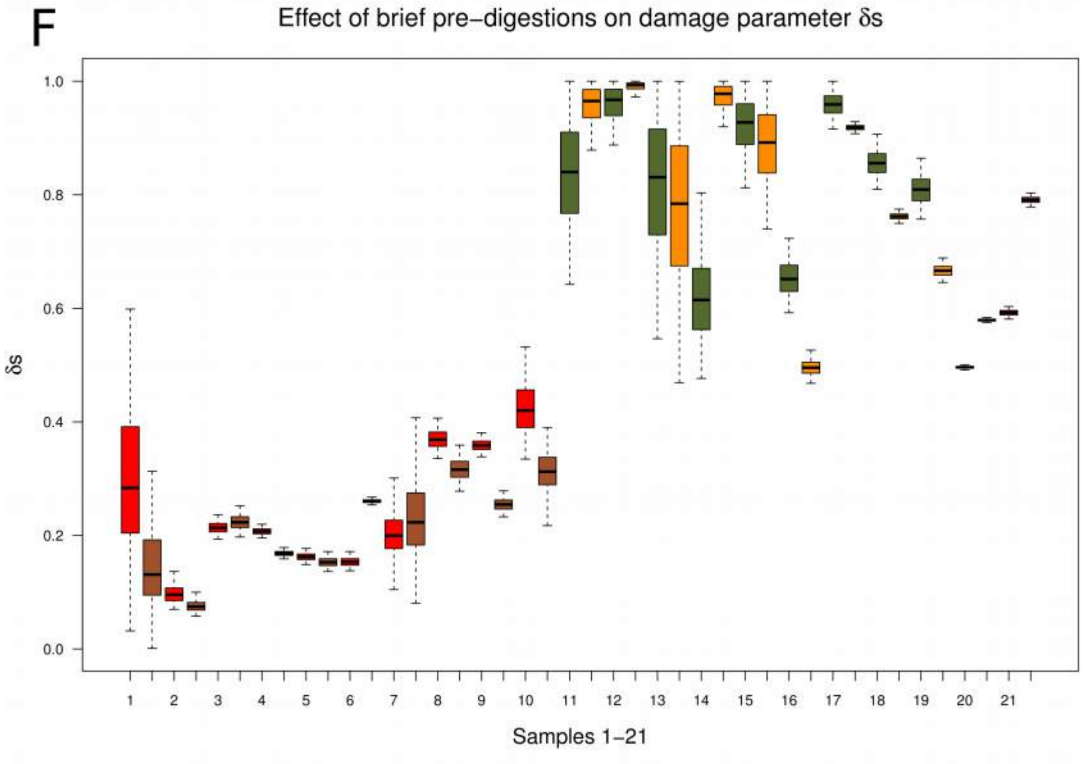
Effect of brief pre−digestions on damage parameter δs
δs
Samples 1−21

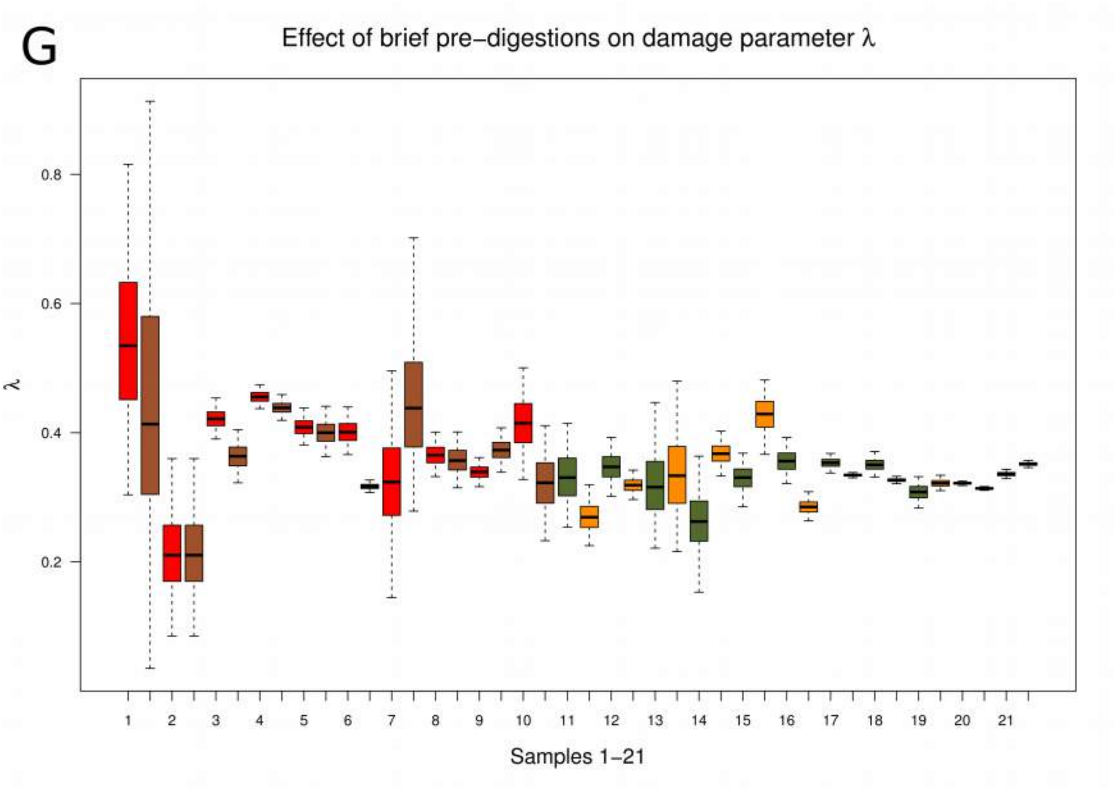

Supplementary Figure S6: A:E Distributions of damage parameter probabilities of δs (deamination rate of cytosine in single stranded context) and of λ (the probability of a base being positioned within a single-stranded overhang) estimated with the Bayesian approach implemented in mapDamage 2.0[3] with standard parameters (ie. 50 000 iterations) obtained on DNA sequences identified as human from **(A)** bone EI8, **(B)** bone EI9, **(C)** bone EI19, **(D)** bone EI22, **(E)** bone Trinitatis. The boxplots display the median value (black stripe), first and third quartile (length of box is the interquartile range), and the IQR is extended 1.5 times with the whiskers. In **(F)** the distributions of the damage parameter probabilities for δs performed on non-predigested (red and green) and briefly pre-digested (15-30 min) (brown and yellow) are similarly plotted. In **(G)** the distributions of the damage parameter probabilities of λ performed on non-predigested (red and green) and briefly pre-digested (15-30 min) (brown and yellow) are similarly plotted. For **(F)** and **(G)** samples in red and brown were amplified with TaqGold. Samples in green and yellow were amplified with Kapa U+. Samples 1-21 refer to 1) ANR 4704, 2) ANR 4705, 3) ANR 4706, 4) ANR 4707, 5) ANR 4708, 6) ANR 4709, 7) ANR 4710, 8) ANR 4712, 9) ANR 4714, 10) ANR 4715, 11) AAG1, 12) AAG2, 13) AAG3, 14) AAG4, 15) AAG5, 16) AAG6, 17) AAG7, 18) AAG8, 19) AAG9, 20) Rise 479, 21)Rise 483

2 Supplementary Methods S1

Library preparation

A volume of 20 µL of DNA extract was used for each library, without prior nebulization as aDNA is already highly fragmented. The DNA was incubated with an end-repair buffer mix containing dNTPs and enzymes that removes the 3' end overhangs, synthesizes complementary strands to the 5'end overhangs, leaving the DNA blunt-ended with 5' end phosphates and 3' end hydroxyls. The reaction was performed in a total of 25 µL for 20 minutes at 12°C, and 15 minutes at 37°C. The blunt-end DNA was purified in 15 µL EB Buffer using Qiagen MinElute silica spin-columns, with 10x volume manufactured PB buffer as binding buffer for samples except AAG1 to AAG9 for which 10x PN Buffer was used as binding buffer. In a volume of 25 µL, Illumina adaptors were ligated to the DNA 5' end, and a Bst Polymerase was used to fill in the sequence between the 3'end adaptors and the inserted DNA (New England Biolabs Inc., Manual Version 2.1).

Library amplification

The following PCR setup and conditions were used for all library amplifications except for AAG1-9: amplifications undertaken in 50 µL mixes, using 5 µL 10X PCR buffer, 4 µL $MgCl_2$ (50 mM), 1 µL BSA (20 mg/ml), 0.5 µL dNTPs (25 mM), 1 µL of each primer (10 µM, inPE forward primer: (5’- AATGATAC GGCGACCACCGAGATCTACACTCTTTCCCTACACGACGCTCTTCCGATCT) + custom indexed reverse primer (5’- CAAGCAGAAGA CGGCATACGAGATNNNNNNGTGACTGGAGTTC)), and 1 µL AmpliTaq Gold DNA Polymerase (Applied Biosystems). The first amplification was carried out with initial 5 minutes at 94°C, followed by a 12 cycles of 30 seconds at 94°C, 30 seconds at 60°C and 40 seconds at 72°C, ultimately with a 7 minute elongation step at 72°C. Identical conditions were

used for the second amplification, with a slightly different PCR mix consisting of 2.5 µl 10X PCR buffer, 2 µl MgCl2 (50 mM), 0.5 µl BSA (20 mg/ml), 0.25 µl dNTPs (25 mM), 1 µl of each primer (10 µM, P5 and P7), and 0.5 µl AmpliTaq Gold DNA Polymerase, 5 µL of first amplified library and 12 µL of molecular grade water, and using 8-12 cycles in the amplification steps. The amplified library was purified with PB buffer on Qiagen MinElute columns, before being eluted in 30 µl EB.

For samples AAG1-9, 10 µL of the DNA extracts were amplified and indexed in a 50 µl PCR reactions, containing 1X KAPA HiFi HotStart Uracil+ ReadyMix (KAPA Biosystems, Woburn, MA, USA) and 200 nM of each of Illumina's Multiplexing PCR primer inPE1.0 and a custom-designed index primer with a six nucleotide index. Thermocycling conditions were as follows: 1 min at 94°C, followed by 8-16 cycles of 15 sec at 94°C, 20 sec at 60°C, and 20 sec at 72°C, and a final extension step of 1 min at 72°C. The optimal number of cycles was determined by qPCR, as done in Kircher et al. 2010. The amplified libraries were then purified using Agencourt AMPure XP beads (Beckman Coulter, Krefeld, Germany).

References

1. Deagle, B. E., Eveson, J. P. & Jarman, S. N. Quantification of damage in DNA recovered from highly degraded samples–a case study on DNA in faeces. *Front. Zool.* **3,** 11 (2006).
2. Allentoft, M. E. *et al.* The half-life of DNA in bone: measuring decay kinetics in 158 dated fossils. *Proc. R. Soc. B Biol. Sci.* **279,** 4724–4733 (2012).
3. Jónsson, H., Ginolhac, A., Schubert, M., Johnson, P. L. & Orlando, L. mapDamage2. 0: fast approximate Bayesian estimates of ancient DNA damage parameters. *Bioinformatics* btt193 (2013).
